# A methodology for the selection and characterization of riboflavin-overproducing *Weissella cibaria* strains after treatment with roseoflavin

**DOI:** 10.3389/fmicb.2023.1154130

**Published:** 2023-04-06

**Authors:** Iñaki Diez-Ozaeta, Lucía Martín-Loarte, Mari Luz Mohedano, Mercedes Tamame, José Ángel Ruiz-Masó, Gloria del Solar, María Teresa Dueñas, Paloma López

**Affiliations:** ^1^Departamento de Biotecnología Microbiana y de Plantas, Centro de Investigaciones Biológicas Margarita Salas (CSIC), Madrid, Spain; ^2^Departamento de Química Aplicada, Facultad de Química, Universidad del País Vasco (UPV/EHU), San Sebastián, Spain; ^3^Instituto de Biología Funcional y Genómica, (IBFG) CSIC-Universidad de Salamanca, Salamanca, Spain

**Keywords:** *Weissella cibaria*, lactic acid bacteria, vitamin B2 (riboflavin), FMN riboswitch, roseoflavin, riboflavin overproducing bacteria, regulation of rib operon

## Abstract

Fermentative processes by lactic acid bacteria can produce metabolites of interest to the health and food industries. Two examples are the production of B-group vitamins, and of prebiotic and immunomodulatory dextran-type exopolysaccharides. In this study, three riboflavin- and dextran-producing *Weissella cibaria* strains (BAL3C-5, BAL3C-7 and BAL3C-22) were used to develop a new method for selection and isolation of spontaneous riboflavin-overproducing *W. cibaria* mutants. This method was based on the selection of strains resistant to roseoflavin. The DNA sequencing of the FMN riboswitch of bacterial cell populations treated with various roseoflavin concentrations, revealed the existence of at least 10 spontaneous and random point mutations at this location. Folding and analysis of the mutated FMN riboswitches with the RNA fold program predicted that these mutations could result in a deregulation of the *rib* operon expression. When the roseoflavin-treated cultures were plated on medium supporting dextran synthesis, the most promising mutants were identified by the yellow color of their mucous colonies, exhibiting a *ropy* phenotype. After their isolation and recovery in liquid medium, the evaluation of their riboflavin production revealed that the mutant strains synthesized a wide range of riboflavin levels (from 0.80 to 6.50 mg/L) above the wild-type level (0.15 mg/L). Thus, this was a reliable method to select spontaneous riboflavin-overproducing and dextran-producing strains of *W. cibaria.* This species has not yet been used as a starter or adjunct culture, but this study reinforces the potential that it has for the food and health industry for the production of functional foods or as a probiotic. Furthermore, analysis of the influence of FMN present in the growth medium, on *rib* mRNA and riboflavin levels, revealed which mutant strains produce riboflavin without flavin regulation. Moreover, the BAL3C-5 C120T mutant was identified as the highest riboflavin-overproducer. Determination of its chromosomal DNA sequence and that of BAL3C-5, revealed a total identity between the 2 strains except for the C120T mutation at the FMN riboswitch. To our knowledge, this work is the first demonstration that only a single alteration in the genome of a lactic acid bacteria is required for a riboflavin-overproducing phenotype.

## 1. Introduction

Lactic acid bacteria (LAB) have the metabolic capacity to synthesize B-group vitamins and dextran-type exopolysaccharides (EPS), which have a wide range of functionalities and properties. Dextran is an α-glucan polysaccharide mainly composed of D-glucopyranosyl residues with α-(1,6) linkages and varying percentages of α-(1,4), α-(1,3) or α-(1,2) branches (Bounaix et al., 2010). It is synthesized extracellularly by diverse LAB in a reaction catalyzed by dextransucrases (Dsr, enzymes belonging to the glycoside hydrolase GH 70 family) by hydrolysis of sucrose and transfer of glucose molecules to the growing chain of the polymer. Dextrans are potential new hydrocolloids with interesting rheological properties, improving the structure/texture of different foods (e.g., in the formulation of gluten-free bakery or low-fat dairy products; Lynch et al., 2018; Werning et al., 2022). In addition, the high molecular weight dextran produced by LAB strains could have also multiple beneficial properties for human health, since they can act as antiviral (Nácher-Vázquez et al., 2015), antioxidant, hypocholesterolemic (Nadzir et al., 2021) and prebiotic (Kim et al., 2022) agents. Moreover, they have shown immunomodulatory (Zarour et al., 2017), and anti-inflammatory (Soeiro et al., 2016; Zhou et al., 2022) properties.

Riboflavin (RF, vitamin B_2_) is a water-soluble vitamin that is part of the vitamin B complex. It is the precursor of both flavin adenine dinucleotide (FAD) and flavin mononucleotide (FMN), which are essential coenzymes in many oxidation–reduction processes and play an important role in cell energy metabolism, and therefore is an essential micronutrient for human health and development. RF is not synthetized by the human body, and it must be obtained from ingested food and/or from the gut microbiota (Leblanc et al., 2015; Thakur et al., 2017). RF is mainly found in foodstuff of animal origin such as meat, eggs and dairy products, and in lower concentrations in legumes, cereals or other vegetables. The daily intake recommended by EFSA ranges from 0.3–1.5 mg/day depending on the population group (EFSA Panel on Dietetic Products, Nutrition and Allergies (EFSA NDA Panel), 2017). It is mainly absorbed in the proximal small intestine and excreted in the urine, and its deficiency is due to malabsorption or insufficient vitamin intake. RF deficiency (ariboflavinosis) is of worldwide concern. Although this health issue is common in developing countries, RF deficiency also occurs in developed countries, mainly in populations with low intake of animal origin foodstuff (vegans/vegetarians) or with a greater need for RF intake due to their physiological condition (pregnant women, young people or elderly; Gregory et al., 2000; Rohner et al., 2007; Titcomb and Tanumihardjo, 2019). Its deficiency can lead to various health disturbances, including migraine, cardiac and skin disorders or alterations in sugar metabolism. It plays a key role in the homeostasis of the human body (Dey and Bishayi, 2016), regulation of multiple metabolic pathways driven by redox reactions (Powers, 2003) or the metabolism of different vitamins (such as folic acid, niacin, pyridoxine, and cobalamin) through the action of FMN and FAD (Pinto and Rivlin, 2013). Besides its important role in maintaining human health, recently the antimicrobial activity of RF against parasites, fungi, viruses and bacteria has been demonstrated (Farah et al., 2022). Therefore, *in situ* biofortification of fermented foods through the use of LAB that overproduce vitamin B_2_ and dextran-type EPS, is a promising strategy to strengthen the health of consumers and address different nutritional deficiencies.

With regard to large scale production of riboflavin, the biosynthesis by microbial fermentation is the most promising and the currently the best candidates as cell factories, beside some fungi, are a few bacteria including *Bacillus subtilis,* LAB and *Escherichia coli* (Zhang et al., 2021).

**Figure 1:**
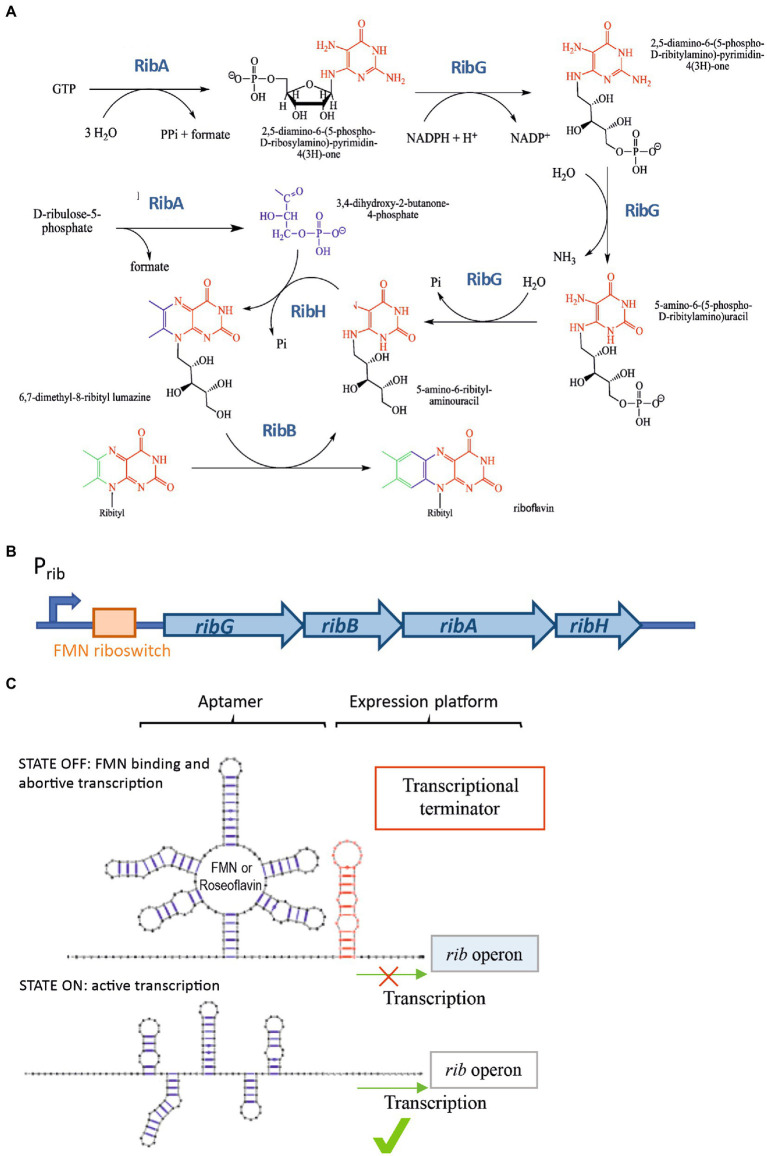
RF synthesis and regulation. **(A)** RF biosynthetic pathway. **(B)** The *rib* operon and its regulatory regions (promoter and FMN riboswitch). **(C)** Schematic representation of the *rib* operon riboswitch including the FMN binding sensing aptamer and the expression platform. Two alternative conformations of the regulatory domain. “OFF state” or FMN-bound state facilitating the formation of a ρ-independent transcriptional terminator in the regulatory element in the RNA, and the “ON state” in which an anti-terminator structure is formed in the absence of FMN enabling the transcription of the *rib* operon.

In most Gram-positive bacteria, including *Bacillus subtilis* and LAB, the synthesis of riboflavin is catalyzed by four proteins: RibG, RibB, RibA, and RibH (Figure 1A), whose coding genes constitute the *rib* operon (Figure 1B). The expression of this operon is regulated by transcriptional attenuation through the FMN riboswitch (also called the RFN element) located in the 5′-untranslated region of the *rib* mRNA (Figure 1C), The riboswitch contains the FMN-binding aptamer, thus, when the concentration of FMN in the bacterial cytosol reaches the necessary level for its role as cofactor, the compound binds to the riboswitch (Vitreschak et al., 2002; Winkler et al., 2002). This binding leads to the formation of a terminator hairpin (in the expression platform of the riboswitch) and repression of transcription occurs, inhibiting the synthesis of RF (Figure 1C). By contrast, in the absence of the flavin, transcription of the operon takes place (Abbas and Sibirny, 2011; Thakur et al., 2015; Figure 1C). This regulatory mechanism is conserved in many distinct species such as *Fusobacterium nucleatum, Bacillus subtilis, Lactococcus lactis, Propionibacterium freudenreichii*, *Leuconostoc mesenteroides,* and *Lactiplantibacillus plantarum* (Burgess et al., 2004, 2006; Serganov et al., 2009; Ripa et al., 2022).

Roseoflavin, is a toxic analog of RF that also has the capacity to interact with the FMN-binding aptamer provoking bacterial death, and therefore treatment with this compound has been classically used to discover riboflavin-overproducing LAB strains. Thus, this procedure has been successfully employed to select spontaneous roseoflavin-resistant and RF-overproducing LAB belonging to the *L. lactis, L. plantarum, Limosilactobacillus reuteri,* and *L. mesenteroides* species (Burgess et al., 2004, 2006; Capozzi et al., 2011; Russo et al., 2014; Mohedano et al., 2019; Ge et al., 2020; Kim et al., 2021). In all these cases, LAB were treated with roseoflavin by: (i) plating in the presence of a high concentration of the compound or (ii) successive exposures to increasing concentrations of the RF analog in liquid medium. In all cases DNA sequencing revealed changes in the upstream region of the *rib* operon. In the case of pickle-derived *L. plantarum*, Ge et al. (2020) found an insertion of 1,059-bp DNA fragment located between the FMN riboswitch and the ribosomal binding site of the first gene of the *rib* operon, which could be responsible for alterations in the *rib* operon expression. Besides that, in all the other cases, including *L. plantarum* roseoflavin-treated strains isolated from various habitats, point mutations or deletions in the FMN riboswitch were observed.

In addition, we have isolated from rye sourdough three *Weissella cibaria* strains (BAL3C-5, BAL3C-7, and BAL3C-22) which are able to produce dextran and RF (Llamas-Arriba et al., 2021), and selected three RF-overproducing mutants, each from one of the above parental strains, by treatment with increasing concentrations of roseoflavin (Hernández-Alcántara et al., 2022). Moreover, analysis of the mutants´ performance during experimental bread making revealed that indeed they were able to biofortify the bread with dextran and RF by *in situ* synthesis (Hernández-Alcántara et al., 2022). Therefore, these results indicated the potential interest of *W. cibaria* RF-overproducing strains for production of functional bread.

Against this background we here report a strategy for *in vitro* detection of spontaneous mutants, prior to their isolation, in roseoflavin-treated cultures of the previously characterized BAL3C-5, BAL3C-7, and BAL3C-22 strains and their further characterization. Prior to strain selection and isolation, molecular analyses and predictions of the consequences of point mutations in the regulatory region of roseoflavin treated cultures were carried out. Thereby, 8 mutants of interest were isolated, the changes in their FMN riboswitch characterized and their ability for RF overproduction validated under laboratory growth conditions. The influence of FMN and RF supplementation in the growth medium on RF production was also analyzed, as well as the effect of the FMN on the expression of the *rib* operon. Finally, determination of the DNA sequence of the genome of the parental BAL3C-5 strain and of its isogenic RF-overproducing mutant BAL3C-5 C120T revealed that indeed the mutation detected in the FMN-riboswitch was the only difference between the two chromosomal sequences. Thus, as far as we know, this is the first direct demonstration for a LAB strain that, in addition to the single mutation in the FMN riboswitch, no other molecular changes are required for its overproduction of RF.

## 2. Materials and methods

### 2.1. Bacterial strains and growth conditions

*Weissella cibaria* BAL3C-5, BAL3C-7, BAL3C-22 strains previously isolated from rye sourdough (Llamas-Arriba et al., 2021) and designated as parental or wild type (wt) as well as their corresponding RF-overproducing strains were used in this study and their characteristics are described in Table 1. The bacteria were grown at 30°C and propagated in liquid MRS medium (de Man et al., 1960) supplemented with either 2% sucrose (MRSS) or 2% glucose (MRSG). Also, the RF Assay Medium (RAM, Difco) containing 2% glucose, RAM supplemented with 2% sucrose (RAMS) or 2% maltose (RAMM) were used for the bacterial growth, when production of RF and dextran were investigated or during wt strain treatment with roseoflavin. Furthermore, RAMS plus 3 μM RF (RAMS + RF) and RAMS plus 3 μM FMN (RAMS + FMN) media were used to evaluate the influence of the presence of flavins during growth on RF production and *rib* operon expression.

**Table 1:** Bacterial strains used in this work.

*Weissella cibaria* strains	Characteristics	Source of isolation	FMN riboswitch	Reference
BAL3C-5	Riboflavin- and dextran-producer	Fermented rye dough	Wild-type	Llamas-Arriba et al. (2021)
BAL3C-7	Riboflavin- and dextran-producer	Fermented rye doughsdd	Wild-type	Llamas-Arriba et al. (2021)
BAL3C-22	Riboflavin- and dextran-overproducer	Fermented rye dough	Wild-type	Llamas-Arriba et al. (2021)
BAL3C-5 G15T (previously called BAL3C-5 B2)	Riboflavin-overproducer and dextran-producer	Spontaneous mutant of BAL3C-5 selected by roseoflavin treatment	G15T mutant	Hernández-Alcántara et al. (2022)
BAL3C-5 ΔG15	Riboflavin-overproducer and dextran-producer	Spontaneous mutant of BAL3C-5 selected by roseoflavin treatment	ΔG15 mutant	This work
BAL3C-5 A59C	Riboflavin-overproducer and dextran-producer	Spontaneous mutant of BAL3C-5 selected by roseoflavin treatment	A59C mutant	This work
BAL3C-5 A115G	Riboflavin-overproducer and dextran-producer	Spontaneous mutant of BAL3C-5 selected by roseoflavin treatment	A115G mutant	This work
BAL3C-5 C120T	Riboflavin-overproducer and dextran-producer	Spontaneous mutant of BAL3C-5 selected by roseoflavin treatment	C120T mutant	This work
BAL3C-7 G14T	Riboflavin-overproducer and dextran-producer	Spontaneous mutant of BAL3C-7 selected by roseoflavin treatment	G14T mutant	This work
BAL3C-7 G109A (previously called BAL3C-7 B2)	Riboflavin-overproducer and dextran-producer	Spontaneous mutant of BAL3C-7 selected by roseoflavin treatment	G109A mutant	Hernández-Alcántara et al. (2022)
BAL3C-22 C23T (previously called BAL3C-22 B2)	Riboflavin-overproducer and dextran-producer	Spontaneous mutant of BAL3C-22 selected by roseoflavin treatment	C23T mutant	Hernández-Alcántara et al. (2022)

### 2.2. Detection and isolation of RF-overproducing strains

The three parental *W. cibaria* strains were grown in MRSG medium to an optical density at 600 nm (OD_600 nm_) of 2.0. Afterwards, the bacterial cultures were diluted 1:100 in RAM medium and grown to mid exponential phase (OD_600 nm_ of 1.0). Then, the bacterial cultures were diluted in RAMM medium to give an OD_600 nm_ of 0.025 and four aliquots of 1 ml were supplemented each with a different roseoflavin concentration (100, 200, 300, or 400 μg/mL) and further grown at 30°C during approximately 60 h. Afterwards, cultures were sedimented by centrifugation at 9,300 × *g* for10 min at 4°C. The supernatants were removed, the bacterial cells were washed with phosphate-buffered saline (PBS) pH 7.3 and sedimented as above. After this step the cell pellets used for DNA extraction and further molecular analysis were stored at −20°C, whereas samples used for mutant isolation were resuspended in MRS supplemented with 20% glycerol and stored at −80°C. To isolate the mutants, selected roseoflavin-treated cultures were thawed and plated on MRSS agar plates. After 24 h incubation at 30°C, colonies were phenotypically selected (most of them yellowish), recovered from the plates by growth in MRSS and finally stored in MRS containing 20% glycerol at −80°C, until required.

### 2.3. Genomic DNA extraction, PCR amplification, and DNA sequencing of the FMN riboswitches’ coding sequences

Genomic DNA (gDNA) extraction from *W. cibaria* strains was performed using the Wizard Genomic DNA Purification kit (Promega) following the instructions of the supplier but with modifications at three steps of the recommended protocol: (i) lysis step was carried out in the presence of lysozyme (30 mg/mL) and mutanolysin (25 U), (ii) DNA precipitation was performed with isopropanol and in the presence of Pellet Paint Coprecipitant NF (Merck), and (iii) after the isopropanol precipitation and supernatant removal, the washing step was performed through capillarity prior to drying the pellet with a vacuum pump and resuspension of the gDNA in 10 mM Tris HCl pH 8.0. DNA integrity was checked by electrophoresis in a 0.8% agarose gel with Tris-Acetate-EDTA buffer (Sigma-Aldrich) containing GelRed (Biotium). After that, gDNA was used as the template for amplification of the *W. cibaria* FMN riboswitch by the polymerase chain reaction (PCR).

PCR reactions were performed following the protocol of the recombinant Taq DNA polymerase (Thermo Fisher Scientific) in a final volume of 50 μL containing: 1x PCR buffer, 1.5 mM MgCl_2_, 0.2 mM dNTPs mix, 0.5 μM from each of the primers, 1–500 ng of the DNA template, 1.0–2.5 U of the Taq DNA polymerase. The primers used were: ForRibo and ReverseRibo (Table 2). The reaction product was a 435 bp fragment including the *rib* operon regulatory region. PCR conditions were as follows: preheating at 94°C for 3 min, 15 PCR cycles of denaturing at 95°C for 45 s, annealing at 59°C for 30 s, extension at 72°C for 90 s and final extension for 10 min. The correct amplification was verified by analysis of the amplicons in 0.8% agarose gel and photographed using a Gel Doc 2000 Bio-Rad gel documentation system (Bio-Rad) and the Quantity One 4.5.2 Bio-Rad software. PCR products were purified with QIAquick PCR purification kit (Qiagen) and then, automated sequencing was performed through Sanger sequencing by Secugen (Madrid, Spain). The obtained sequences were analyzed with Chromas 2.6.6 (Technelysium Pty. Ltd.) and DNASTAR (Lasergene) software. The three parental strains (BAL3C-5, BAL3C-7, BAL3C-22) each carry an identical FMN riboswitch (Hernández-Alcántara et al., 2022) and its DNA sequence was compared with those of the roseoflavin treated cultures and isolates using the BLASTn software (Altschul et al., 1990). Predictions of the secondary structures of the FMN riboswitches were obtained by using the RNAfold web server (The ViennaRNA Web Services, version 2.4.18) and edited with VARNA 3.9 software (Darty et al., 2009).

**Table 2:** Primers used within the study.

Primers for amplification and sequencing of the FMN riboswitch		
Primer name	Primer sequence	Amplicon size (bp)
ForRibo	5´-GAAGTACCGGTATGACTGCTTT-3′	435
RevRibo	5´-TGGTTTCCCCTAACTACTACTCCGG-3′	
Primers for RT-PCR analyses		
Primer name	Primer sequence	Amplicon size (bp)
For1	5´-CCGGAGTAGTTAGGGGAAACA-3′	239
Rev1	5´-GACATACATCGTGGCCCCAA-3′	
For2	5´-GAAGTACCGGTATGACTGCTTT-3′	214
Rev2	5´-TCAACCGAATTGCTTAATCGCA-3´	
For*rpoB*	5´-GTCCATCAATGGAGCAAGGT-3′	224
Rev*rpoB*	5´-TAAACATCATCGCGGATCAA-3´	

### 2.4. Analysis of bacterial growth as well as RF and dextran production

Overnight cultures of the *W. cibaria* strains grown in MRSG were centrifuged at 9,300 × *g* for 10 min and the cells resuspended in fresh RAMS medium to give an OD_600 nm_ of 0.1. Then, cultures were grown to an OD_600 nm_ of 1.0, sedimented as above and resuspended in either RAMS + RF or RAMS + FMN. Aliquots of 200 μL in triplicate of each culture were analyzed in a sterile 96-well polystyrene optical bottom plate (Thermo Fisher Scientific). Bacterial growth (OD_600 nm_) and fluorescence was monitored in real time, with measurements at 30 min intervals, at 30°C for 16 h using a Varioskan Flask System (Thermo Fisher Scientific), as previously described (Mohedano et al., 2019). RF fluorescence was measured using an excitation wavelength of 440 nm and detection of emission wavelength at 520 nm. RF concentration was calculated using a calibration curve (Mohedano et al., 2019). The growth rate and the doubling time of the strains was determined as previously described (Widdel, 2010).

Also, *W. cibaria* strains were inoculated in 5 ml of either RAMS or RAM to an OD_600nm_ of 0.1 and grown for 16 h at 30°C. Then, the concentration of RF was determined by measuring, as above: (i) the total fluorescence of the cultures and (ii) the fluorescence present in the culture supernatants after cell removal by centrifugation. Moreover, the dextrans present in the culture supernatants were recovered by ethanol precipitation as previously described (Besrour-Aouam et al., 2021) and their concentration determined by the phenol-sulfuric method (Dubois et al., 1956). Quantification was performed using a glucose calibration curve as a standard. Determinations were performed in triplicate.

### 2.5. Quantitative reverse transcription PCR analysis of expression of the *ribG* gene and the FMN riboswitch

Cultures of 5 mL of all *W. cibaria* strains were grown in RAMS or RAMS + FMN medium at 30°C in triplicate. Bacteria were grown until the cultures reached an OD_600nm_ of 1.0. Then, RNA was rapidly stabilized by the addition of RNA Protect Bacteria Reagent (Qiagen) and cells were sedimented at 5,000 x *g* for 5 min. In parallel, aliquots of 1 mL cultures were withdrawn and used to determine the total RF concentration using the Varioskan equipment as described above.

To obtain total RNA, frozen bacterial cells were thawed, lysed by lysozyme (30 mg/mL) and mutanolysin (25 U) treatment, and further thermally disrupted at 80°C for 5 min. Total RNA was then purified following the instructions of the RNeasy Plus Mini kit, which includes on-column gDNA removal (Qiagen). Concentration and purity of extracted RNA was determined with a Nanodrop2000c spectrophotometer (ThermoFisher Scientific) and the integrity was confirmed through gel electrophoresis. Before cDNA synthesis, RNA was treated with ezDNAse (Invitrogen) to remove residual gDNA. Then, a 1 μg of RNA sample was used for cDNA synthesis which was carried out with the SuperScript IV First-Strand Synthesis System kit (Thermo Scientific) following the manufacturer’s instructions. mRNA expression was monitored by real time qPCR carried out with SYBR Green PCR master mix (Roche Diagnostics) on a Roche LightCycler®96 instrument. The sequence of the primers used and the size of the amplicons generated during the qPCR analysis of the *ribG* gene (For1 and Rev1), the coding region of the FMN riboswitch (For2 and Rev2) and the housekeeping *rpoB* gene (For*rpoB* and Rev*rpoB*) are detailed in Table 2. For both assays, the reaction conditions were performed as follows: 95°C for 3 min followed by 40 cycles of 95°C for 20 s, 54°C for 40 s, and 72°C for 20 s and a dissociation step of 95°C for 10 s, 54°C for 60 s, and 97°C for 1 s. Reactions were performed in triplicate. The mean Cq value of each sample was normalized against the housekeeping *rpoB* gene and the corresponding control (see details in the Results section). The relative gene expression quantification was calculated with the 2^-ΔCT^ method (Livak and Schmittgen, 2001).

### 2.6. Whole genome sequencing, assembly, annotation, and analysis

*Weissella cibaria* BAL3C-5 wt and BAL3C-5 C120T mutant strains were grown in MRS medium at 30°C to an OD_600 nm_ of 1.0. Genomic DNA extraction was performed as previously described using the Wizard® Genomic DNA Purification Kit (Promega). Extracted DNA was purified with NucleoSpin Gel and PCR Clean-up Kit (Macherey-Nagel), DNA concentration and quality was checked with Nanodrop and Qubit 2.0 fluorometer (Invitrogen).

Genome sequencing was achieved at Secugen (Madrid, Spain) combining Illumina Miseq technology with 2 × 150 paired-end reads and Oxford Nanopore MiniION technology. Libraries were prepared with the SQK-LSK109 ligation kit (Nanopore Technologies). A label was added to each sample (barcode) with the native barcoding kit EXP-NBD114 and the libraries were loaded in the flow cell FLO-MIN106 (Nanopore Technologies). The Illumina and Nanopore reads were analyzed with a high quality module (super accurate) of the MinKNOW software. The assembly was performed with Galaxy unicycler 0.5.0. software (Wick et al., 2017). Genome annotation was done with Prokka 1.14.6 tool through the Galaxy web-based platform.^1^ Genome mapping visualization was performed through Proksee bioinformatic tool for genome assembly, annotation and visualization.^2^

The genomes were evaluated for the presence of antibiotic resistance genes using the BLAST and Resistance Gene Identifier (RGI) tools together with the Comprehensive Antibiotic Resistance Database (CARD, https://card.mcmaster.ca/; McArthur et al., 2013; Jia et al., 2017). Moreover, screening of resistance genes, genomic islands and virulence factors was assessed by Island4Viewer software^3^ (Bertelli et al., 2017).

### 2.7. Statistical analysis

RF and dextran production as well as RT-PCR results were tested with one-way ANOVA analysis. A *p*-value ≤ 0.05 was considered significant. For each parental-mutants group, comparisons were computed with Dunnett test (α = 0.05), and the comparison of all the strains together was performed by a Tukey’s test. Means with a different letter are significantly different. All analyses were performed with the R software version 4.1.3 (R Foundation for Statistical Computing, Vienna, Austria).

## 3. Results

### 3.1. A method for specific detection and isolation of RF-overproducing LAB

**Figure 2:**
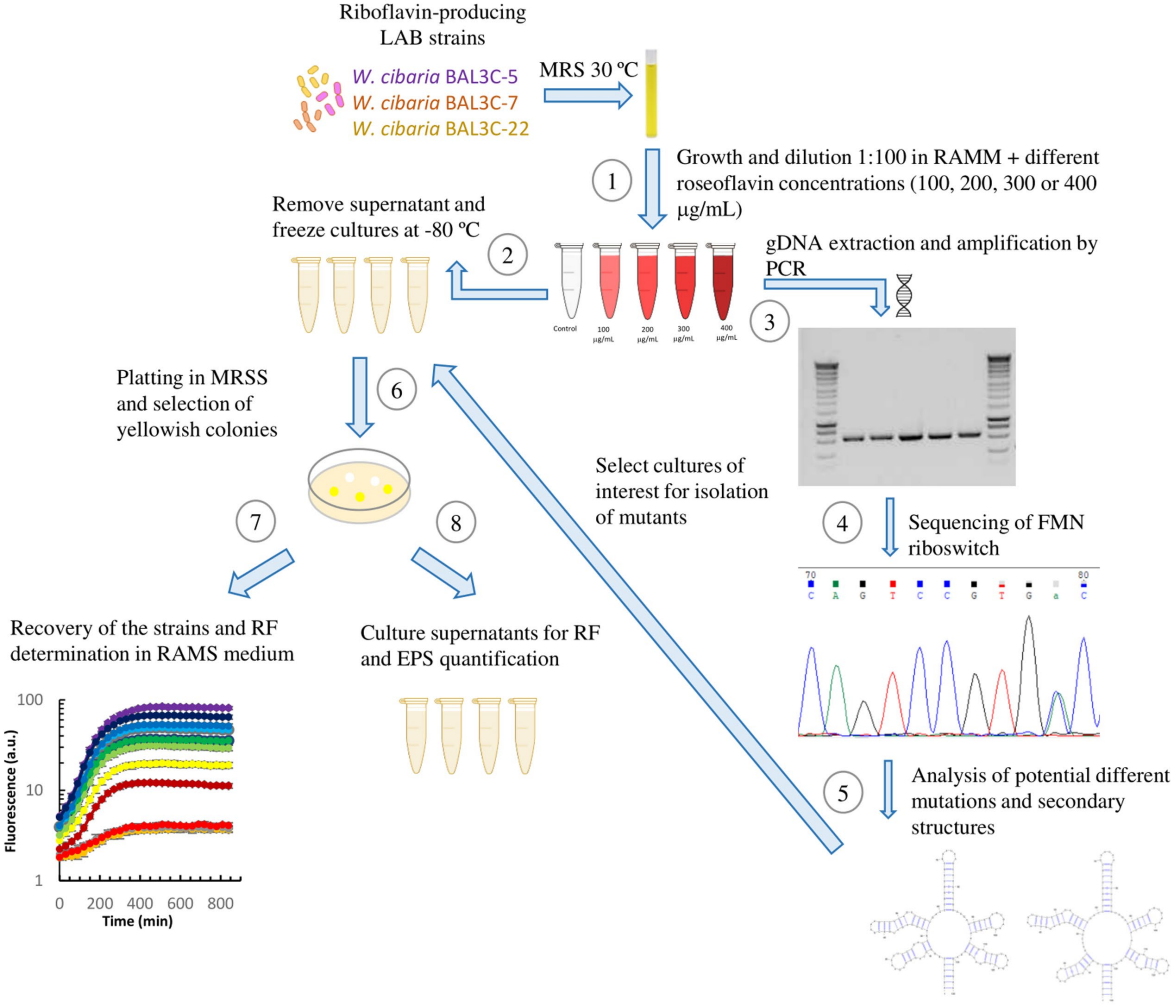
Schematic representation of the methodology followed for the selection and characterization of mutant strains. **(1)** Cultures reconstituted in MRS were grown in RAMM supplemented with different roseoflavin concentrations for 48 h at 30°C. **(2)** Then, aliquots of the cultures were stored at −80°C. Another portion of bacterial cultures was used for gDNA extraction and amplification of the FMN riboswitch region. Amplified sequences were tested in agarose gels **(3)** and submitted to sequencing **(4)**. After, detection and location of point mutations in the FMN riboswitch aptamer by analysis of DNA sequencing chromatograms. **(5)** The predictive mutated aptamer structures were analyzed, and the *Weissella cibaria* roseoflavin-treated cultures, whose DNA showed the most promising mutations, were plated in MRSS and **(6)** yellow colonies were selected for isolation of the RF-overproducing strains. Then, the isolated mutant strains were subjected to analysis of RF production and growth in real time **(7)**, as well as quantification of RF and EPS production after 16 h of growth **(8)**.

A new methodology, including *in vivo*, *in vitro*, and *in silico* experiments and analysis, was devised as depicted in Figure 2.

**Figure 3:**
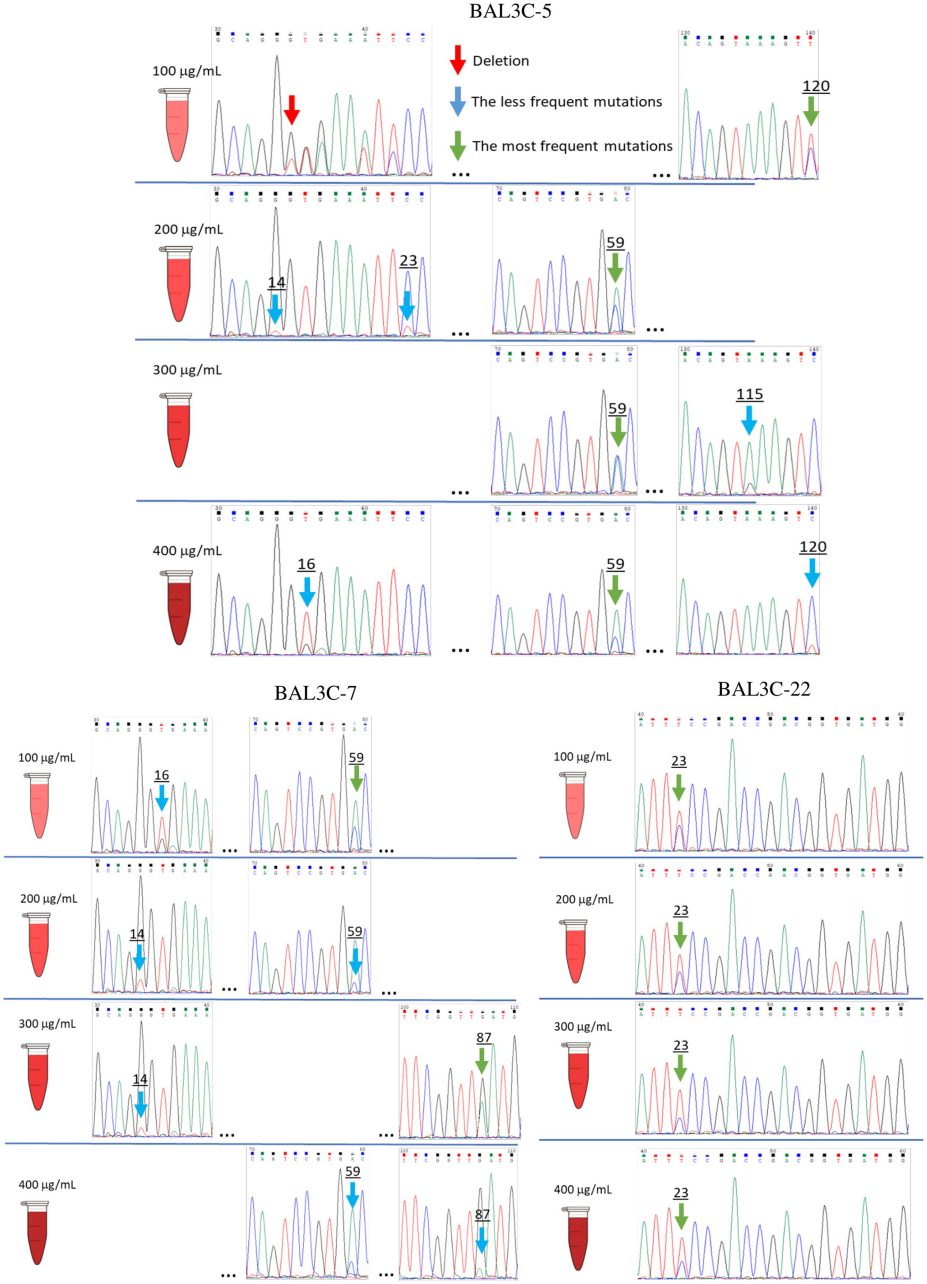
Identification of FMN riboswitch mutations present in roseoflavin treated *Weissella cibaria* cultures. Chromatograms of gDNA sequencing showing the *W. cibaria* BAL3C-5, BAL3C-7, and BAL3C-22 wt and mutants FMN riboswitches. The mutations were detected after various roseoflavin treatments.

Three RF and dextran-producing *W. cibaria* strains (BAL3C-5, BAL3C-7, and BAL3C-22) were used in the present work to detect and isolate RF-overproducing strains. The three strains were independently treated with various concentrations of roseoflavin (100, 200, 300, and 400 μg/mL). Then, prior isolation of mutants by plating and recovering individual colonies, with the aim to detect *in vitro* potential mutations in the bacterial pools, gDNAs of the treated as well as untreated control cultures were extracted, the DNAs encoding the FMN riboswitches of their *rib* operons were amplified and their sequences determined. Some of the chromatograms obtained from the gDNAs analysis are depicted in Figure 3. As expected from previous results (Hernández-Alcántara et al., 2022), the gDNAs from the untreated cultures of three parental strains carried identical FMN riboswitch encoding regions (data not shown). Furthermore, 9 single base substitutions at positions 14, 15, 16, 23, 59, 87, 109, 115, and 120;(the first ribonucleotide of the FMN riboswitch aptamer was considered position 1) and a single-nucleotide deletion at position 15 were detected in the DNA pools of the roseoflavin-treated cultures together with the wt sequence (Figure 3). In addition, minor and predominant mutations could be discerned. Predominant mutations were defined as having an equal or higher frequency than the corresponding wt strain DNA sequence, according to the intensity of their chromatographic peaks. For *W. cibaria* BAL3C-5, mutations with punctual substitutions G14T, G15T, T16G, C23T, A59C, A115G, and C120T, as well as a deletion at position 15 (ΔG15), were detected. The mutations G14T, T16G, A59C, G87A, and G109A were observed in treated cultures of *W. cibaria* BAL3C-7, and finally, a unique mutation C23T was detected in *W. cibaria* BAL3C-22 treated cultures. Moreover, it was also observed that the number and nature of the mutations was independent of the roseoflavin concentration.

**Figure 4:**
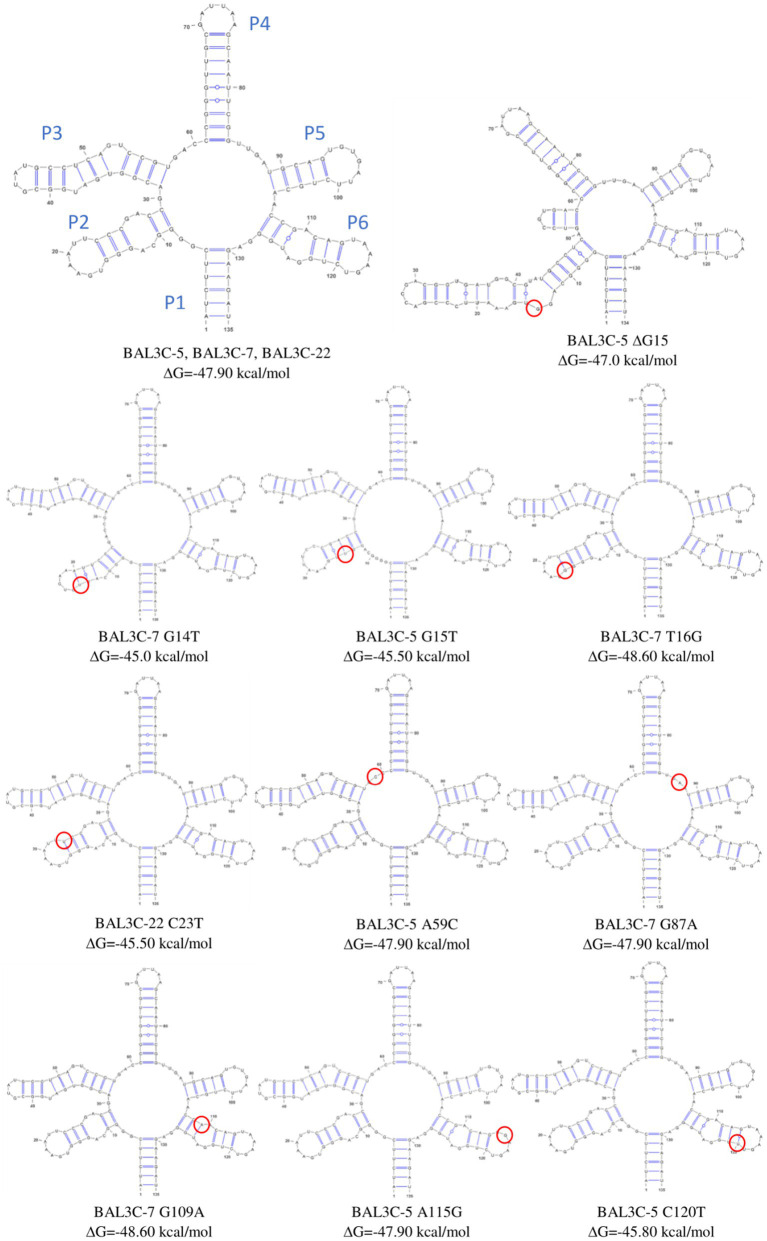
Predictive folding of the FMN riboswitch aptamer of the parental and of all the detected mutant strains. Change in Gibbs free energy (ΔG) for each secondary structure and location of each mutation (red circle) are also shown.

**Figure 5:**
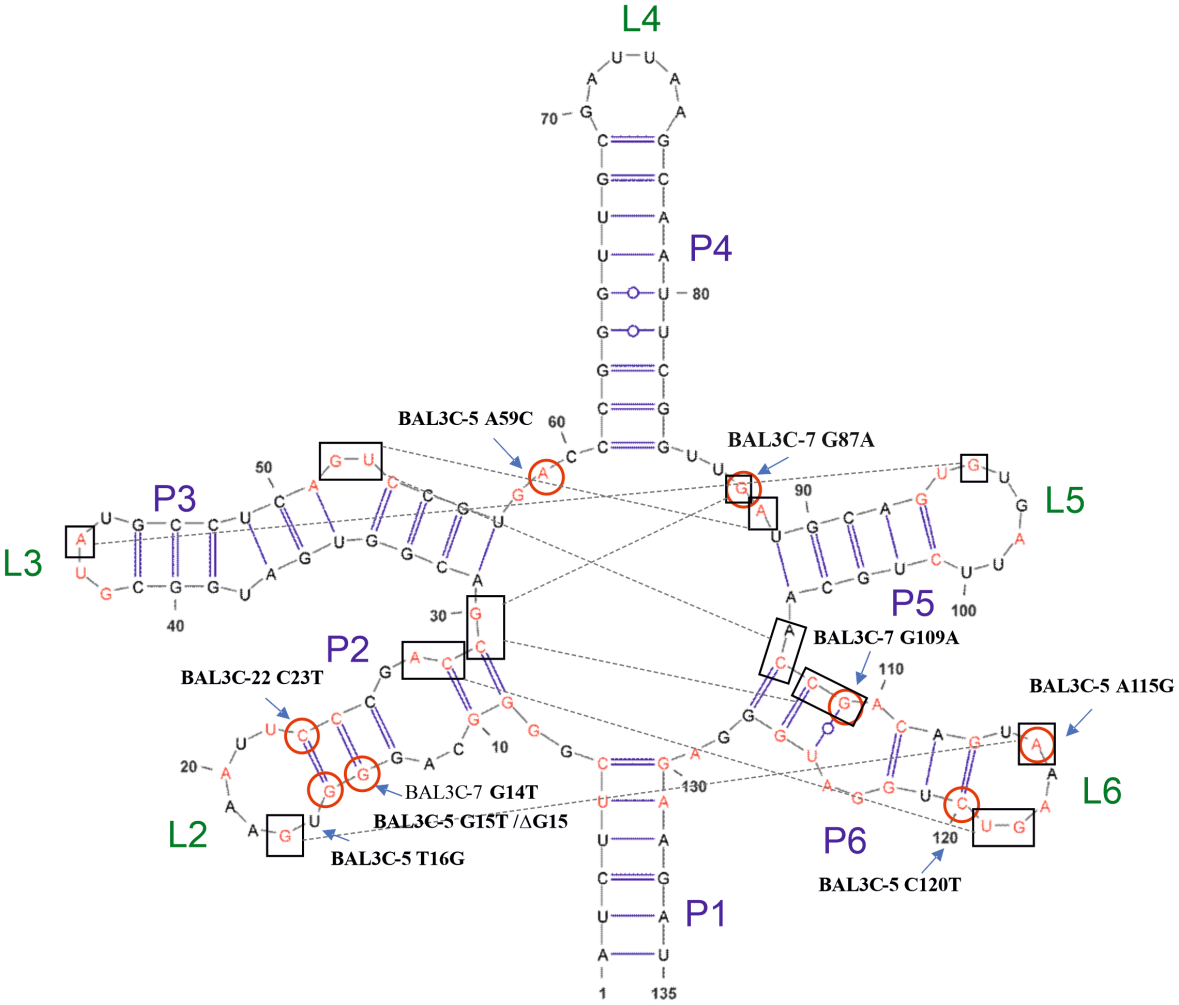
Model of the FMN riboswitch aptamer of *Weissella cibaria* based on the crystallographic studies performed by Serganov et al. (2009). Included in boxes are the nucleotides that could interact which each other for the conformation of the tertiary structure together with the FMN. In red, conserved nucleotides among distant species are depicted. Also, positions of detected mutations are shown.

The aim of this mutagenic analysis was to obtain the mutants with the highest constitutive RF production, and independent of FMN regulation. Therefore, before isolating the mutant strains, an *in silico* analysis of the wt and mutants FMN riboswitches was performed. The RNAfold program was used to predict the folding of the wt and mutants FMN aptamer domain (Figure 4). Moreover, the program allowed the calculation of the Gibbs free energy (ΔG) and the predicted values are also indicated in Figure 4. All the detected mutations were located in the aptamer of the riboswitch and only mutations A59C and G87A were located outside of the stem-loop structures. In addition, in 4 of the aptamers, the mutations (G14T, ΔG15, G15T y T16G) provoked changes in one of the stem loops structures (P2/L2, see details in Figure 5). Regarding to the ΔG required for formation of the FMN riboswitches, the folding of the structures built as a consequence of G14U, G15U, C23U, C120U and ΔG15 mutations showed higher ΔG (−45.0, −45.5, −45.5, −45.8, and − 47.0 kcal/mol, respectively) than that of the wt folding structure (−47.9 kcal/mol). By contrast, the foldings of the A59C, G87A, and A115G aptamers, showed the same predicted ΔG as the wt, and the structure carrying the U16G, and G109A mutations, an even more favorable ΔG (both −48.6 kcal/mol) than the parental structure. With regard to the U16G, and G109A aptamer mutations, their low ΔG are the consequence of the change in the strength of only one base pairing. This takes place by: (i) formation of hydrogen bonds between the G16 and U22 in the T16G mutant not present in the wt, which decreased the size of the P2 loop, and (ii) the interaction of A119 with U125 in the aptamer of the G109A mutant instead of the pairing of G109 with U125 in the structure of the wt strain (Figure 4).

Consequently, all the conformational and ΔG predicted changes of the FMN riboswitch aptamer indicated that the strains carrying most of the detected mutations should have altered regulation of the *rib* operon expression. Therefore, detection and isolation of strains carrying the mutations was approached by plating only the appropriate roseoflavin treated cultures for which the area of the mutated nucleotide was more prominent in the chromatograms, taking advantage of the fact that some RF-overproducing strains are qualitatively detectable by the turning of the colony and/or growth medium from white to yellow, due to the fluorescence of the flavin. However, when some of the treated cultures were plated in MRSG medium, no yellow colonies were detected. Consequently, we took into consideration that due to the production of dextran in MRSS solid medium, *W. cibaria* forms large mucous colonies, in which presumably even a pale yellow color of colonies from low RF-overproducer could be distinguished from the white colonies generated by the wt strains (Supplementary Figure S1). In fact, as part of the methodology used here by plating the selected roseoflavin-treated culture of the BAL3C-22 strain, colonies carrying the C23T mutation could be detected and isolated; strains harboring the G14T or G109A mutations were obtained from BAL3C-7 cultures and the other mutants were obtained from BAL3C-5 treated strain. Only A59C and A115G mutations, produced white colonies in MRSS. Finally, we could not recover the minor T16G and G87A mutations. A picture of liquid cultures of the BAL3C-5, BAL3C-7 and BAL3C-22 parental strains, as well as their mutant derivatives, is shown in Supplementary Figure S1A. A gradation of yellowish color was observed, with that of the BAL3C-5 C120T strain being the more intense. Thus, as an example of color differentiation of colonies, cultures in solid medium of the parental BAL3C-5, and mutant BAL3C-5 C120T strains alone or in a mixed culture are shown in Supplementary Figures S1B–D.

Production of dextran seems to be a common feature of *W. cibaria* species, since more than 50 strains represented in non-redundant protein sequences data bases (NCBI), carry dextransucrase proteins (annotated as glycosyl hydrolases). Therefore, the phenotypical method for selection of RF-overproducing strains by the yellowish color of their colonies in MRSS solid medium could be generally applied to various parental *W. cibaria* strains.

### 3.2. Analysis and quantification of RF production by the *Weissella cibaria* strains

An analysis of the bacterial growth and RF production of the isolated 8 mutants in comparison with their parental strains was performed. A fluorescent method was used to detect the RF production in real time (Mohedano et al., 2019). For this analysis, RAMS medium was used, since it only supports the growth of RF-producing strains, and it is suitable to detect quantitatively production of RF by *W. cibaria* (Llamas-Arriba et al., 2021). To confirm whether the different mutants obtained were RF-overproducers, and whether flavin production was regulated, growth and production of the flavin in RAMS, RAMS + FMN, and RAMS + RF was monitored in real time by measurement the OD_600 nm_ and the fluorescence emitted by the bacterial cultures, respectively.

**Figure 6:**
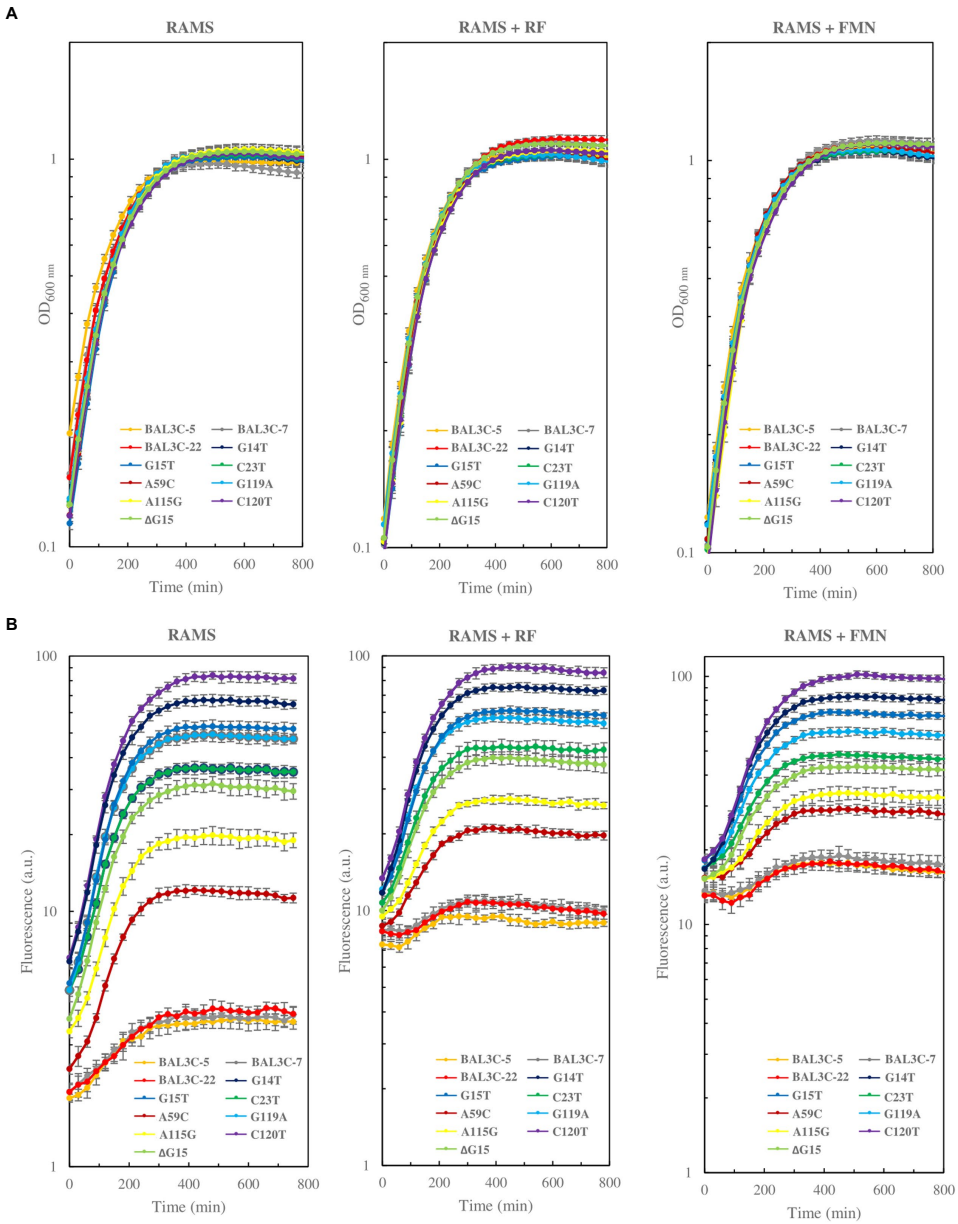
Comparative analysis of growth **(A)** and RF fluorescence **(B)** of the *Weissella cibaria* parental and mutant strains grown in RAMS, RAMS + FMN, and RAMS + RF.

Regarding the growth in each tested medium, all the mutants and the wt strain behaved similarly (Figure 6A). The growth rate and doubling time of the different mutants and their parental strains were very similar. The growth rate ranged from 0.63 to 0.72 h^−1^ in RAMS, between 0.62 to 0.73 h^−1^ in RAMS + FMN, and from 0.69 to 0.77 h^−1^ in RAMS + RF. Regarding the RF production (Figure 6B), the 3 parental strains (BAL3C-5, BAL3C-7, and BAL3C-22) produced low levels of RF in RAMS medium, as previously observed (Llamas-Arriba et al., 2021; Hernández-Alcántara et al., 2022). In addition, all the mutant strains produced different levels of RF, which were higher than those synthesized by the parental strains. BAL3C-5 C120T was the highest producer and BAL3C-5 A59C the lowest. The addition of FMN or RF to the medium altered the basal levels of fluorescence of all strains, and resulted in a pronounced delay in fluorescence increase, ascribed to flavins, only in the cultures of the three parental strains (Figure 6B). In RAMS, RF production by the parental strains was detected from the beginning of growth, whereas in the media containing flavins, the fluorescence did not start to increase until the middle of the exponential phase (Figure 6B). This could be explained by an inhibition of the *rib* operon expression mediated by FMN internalized from the medium or synthesized from internalized RF. By contrast, the presence of either FMN or RF has little or no influence on the behavior of the mutant strains since increase of fluorescence due to the presence of flavins was observed almost from the beginning of the exponential growth phase. Also, the same pattern of RF production was detected among mutant strains.

To further characterize the production of RF by the *W. cibaria* strains, they were grown for 16 h at 30°C in either RAM (containing glucose) or RAMS (containing glucose plus sucrose). The final biomass of the LAB was assessed by plate counting. Their RF production was determined by measurement of: (i) the total fluorescence (total RF) and (ii) the fluorescence of the culture supernatants (free RF; Table 3). All the strains analyzed produced more RF in RAMS than in RAM (Table 3; Supplementary Figures S2–S5), and in addition the behavior of each one of the strains was the same in both media. Parental strains released a low proportion of the vitamin (between 25% and 54% in RAM and around 50% in RAMS) to the supernatant. By contrast, most of the total RF produced by the mutants (more than 90% in RAMS and more than 82% in RAM) was present in the culture supernatant, indicating that these bacteria externalize most of the RF, as previously observed for *L. plantarum* (Mohedano et al., 2019; Ripa et al., 2022). Nevertheless, regarding the RF concentration, wt strains did not show statistically significant differences among them, however, statistically significant differences (*p* < 0.01) were detected among all the mutant strains and between each wt and their mutant derivatives in both growth media tested (Table 3; Supplementary Figures S2–S5). The wt strains were the ones that produce less RF (0.16–0.18 and 0.02–0.03 mg/L in RAMS and RAM, respectively). Among the mutants, RF production by BAL3C-5 A59C was the lowest (1.42 and 0.73 mg/L in RAMS and RAM, respectively) in comparison with the rest of the mutants (Table 3). BAL3C-5 C120T produced the highest levels of total RF production (6.78 mg/L and 5.10 mL/L in RAMS and RAM, respectively). The increase in RF production between the wt and mutant strains was more pronounced in the case of RAM than in RAMS, presumably due to the low RF production observed in RAM for the wt strains. It is worth noting that BAL3C-5 C120T generated after 16 h of growth at 30°C almost 290-fold and 70-fold higher levels of RF than the parental strain in RAM and in RAMS, respectively. Thus, this strain will be the most interesting to be tested in the future for use in functional food production. BAL3C-7 G14T was the second highest RF producer (5.16 and 3.30 mg/L in RAMS and RAM, respectively) and their levels of production were also high compared with other overproducer mutants belonging to other species. Detection of the RF yellow color in strains grown in liquid and solid media also confirmed that BAL3C-5 C120T was the highest producer compared with the rest of the mutants and the wt strains (Supplementary Figure S1). In terms of the biomass in each medium, no significant differences between the wt and the mutant strains were observed. The CFU/L in RAMS ranged between 1.41 × 10^11^ and 2.16 × 10^11^ and in RAM from 8.23 × 10^10^ to 1.16 × 10^11^.

**Table 3:** Analysis of riboflavin and dextran produced by the wt and mutant strains in RAM and RAMS.

Strains	Medium	Total RF (mg/L)^2^	Free RF (mg/L)^2^	Free RF/Total RF (%)	Free RF mutant/Free RF wt	OD_600 nm_	CFU/L	EPS (g/L)^2^	EPS/OD_600 nm_ (g/L)
BAL3C-5	RAMS	0.16 ± 0.02^I^	0.1 ± 0.01^i^	59.66	-	3.23 ± 0.15	1.48E+11	7.02 ± 0.41^αβ^	2.04
BAL3C-7	RAMS	0.18 ± 0.01^I^	0.09 ± 0.01^i^	51.93	-	3.37 ± 0.06	2.11E+11	6.80 ± 0.45^α^	2.02
BAL3C-22	RAMS	0.18 ± 0.02^I^	0.09 ± 0.01^i^	49.52	-	3.27 ± 0.12	1.68E+11	7.10 ± 0.68^αβ^	2.17
BAL3C-5 A59C	RAMS	1.42 ± 0.09^C^	1.28 ± 0.06^c^	90.22	13.25	3.47 ± 0.15	2.16E+11	6.15 ± 0.84^αβ^	1.83
BAL3C-5 A115G	RAMS	2.09 ± 0.12^H^	1.99 ± 0.07^h^	95.29	20.68	3.2 ± 0.10	1.23E+11	6.73 ± 0.57^αβ^	2.10
BAL3C-5 ΔG15	RAMS	2.3 ± 0.08^G^	2.3 ± 0.07^g^	98.70	23.89	3.5 ± 0.20	1.48E+11	6.37 ± 0.78^αβ^	1.82
BAL3C-22 C23T	RAMS	3.25 ± 0.12^E^	3.16 ± 0.10^e^	97.14	36.22	3.4 ± 0.17	1.96E+11	6.01 ± 0.84^αβ^	1.77
BAL3C-7 G109A	RAMS	3.69 ± 0.13^B^	3.52 ± 0.11^b^	95.27	37.77	3.1 ± 0.10	1.94E+11	5.99 ± 0.70^αβ^	1.93
BAL3C-5 G15T	RAMS	4.52 ± 0.21^A^	4.16 ± 0.10^a^	92.07	43.19	3.23 ± 0.15	2.12E+11	6.23 ± 0.82^αβ^	1.81
BAL3C-7 G14T	RAMS	5.16 ± 0.16^D^	4.82 ± 0.17^d^	89.94	51.75	3.4 ± 0.20	1.50E+11	5.60 ± 0.54^β^	1.65
BAL3C-5 C120T	RAMS	6.78 ± 0.13^F^	6.67 ± 0.11^f^	98.33	69.16	3.47 ± 0.12	1.41E+11	6.29 ± 0.70^αβ^	1.81
BAL3C-5	RAM	0.03 ± 0.01^H^	0.02 ± 0.01^h^	53.82	-	2.50 ± 0.10	1.02E+11	^1^n.d	-
BAL3C-7	RAM	0.03 ± 0.01^H^	0.01 ± 0.00^h^	33.46	-	2.47 ± 0.12	9.50E+10	^1^n.d	-
BAL3C-22	RAM	0.04 ± 0.01^H^	0.01 ± 0.01^h^	25.00	-	2.40 ± 0.10	8.23E+10	^1^n.d	-
BAL3C-5 A59C	RAM	0.73 ± 0.04^C^	0.60 ± 0.03^c^	82.23	37.18	2.53 ± 0.06	9.45E+10	^1^n.d	-
BAL3C-5 A115G	RAM	1.08 ± 0.06^G^	0.94 ± 0.03^g^	86.3	57.75	2.67 ± 0.12	9.98E+10	^1^n.d	-
BAL3C-5 ΔG15	RAM	1.46 ± 0.12^D^	1.31 ± 0.07^d^	90.05	80.97	2.53 ± 0.15	9.87E+10	^1^n.d	-
BAL3C-22 C23T	RAM	1.80 ± 0.10^E^	1.52 ± 0.05^e^	84.71	122.24	2.63 ± 0.06	1.05E+11	^1^n.d	-
BAL3C-7 G109A	RAM	2.81 ± 0.16^B^	2.46 ± 0.14^b^	87.65	215.01	2.5 ± 0.17	1.16E+11	^1^n.d	-
BAL3C-5 G15T	RAM	2.85 ± 0.09^F^	2.47 ± 0.11^f^	86.45	152.21	2.53 ± 0.15	9.68E+10	^1^n.d	-
BAL3C-7 G14T	RAM	3.30 ± 0.14^C^	3.11 ± 0.09^c^	94.13	271.23	2.63 ± 0.06	9.83E+10	^1^n.d	-
BAL3C-5 C120T	RAM	5.10 ± 0.16^A^	4.66 ± 0.12^a^	91.28	287.51	2.7 ± 0.10	1.13E+11	^1^n.d	-

1 n.d., non-detected.

2 Different letters mean statistically significant difference (*p* ≤ 0.01).

### 3.3. Dextran production by the *Weissella cibaria* strains

The RF-overproducing phenotype could have a collateral influence in the dextran production of the mutant strains. Therefore, a comparative analysis of EPS production by the LAB strains was performed. The concentration of dextran, present in the supernatants of the parental and mutant strains grown 16 h at 30°C in RAMS, was determined, after ethanol precipitation, by the phenol sulfuric acid method (Table 3; Supplementary Figure S6). As expected, no production was detected in RAM, since it lacks sucrose, the substrate required for dextran synthesis by the dextransucrase. In RAMS all the strains produced similar dextran levels, ranging from 7.10 g/l to 5.60 g/l. The 3 parental strains BAL3C-5, BAL3C-7 and BAL3C-22 were the bacteria that produced the highest EPS yield (7.0, 6.8, and 7.1 g/L, respectively). Focusing on the mutant strains, BAL3C-5 A115G (6.73 g/L) and BAL3C-7 G14T (5.60 g/L) were the highest and the lowest EPS producers, respectively. Statistical analysis revealed that only the mutant BAL3C-7 G14T showed lower dextran production than its parental strain (*p* < 0.05; Table 3; Supplementary Figure S6). Nevertheless, although the mutant strain synthesized lower concentration of dextran than the parental bacteria, the EPS production was still high. Moreover, the above results confirmed that RAMS is suitable for the quantification of both RF and dextran production by *W. cibaria* strains.

Regarding dextran production, the good capability of BAL3C-5, BAL3C-7 and BAL3C-22 to produce dextran, as previously observed (Llamas-Arriba et al., 2021; Hernández-Alcántara et al., 2022), was confirmed here. In addition, no significant differences were detected among parental and mutant strains. Hence, dextran production was maintained in the mutants of interest.

### 3.4. Quantification of *ribG* gene expression in *Weissella cibaria* strains

**Figure 7:**
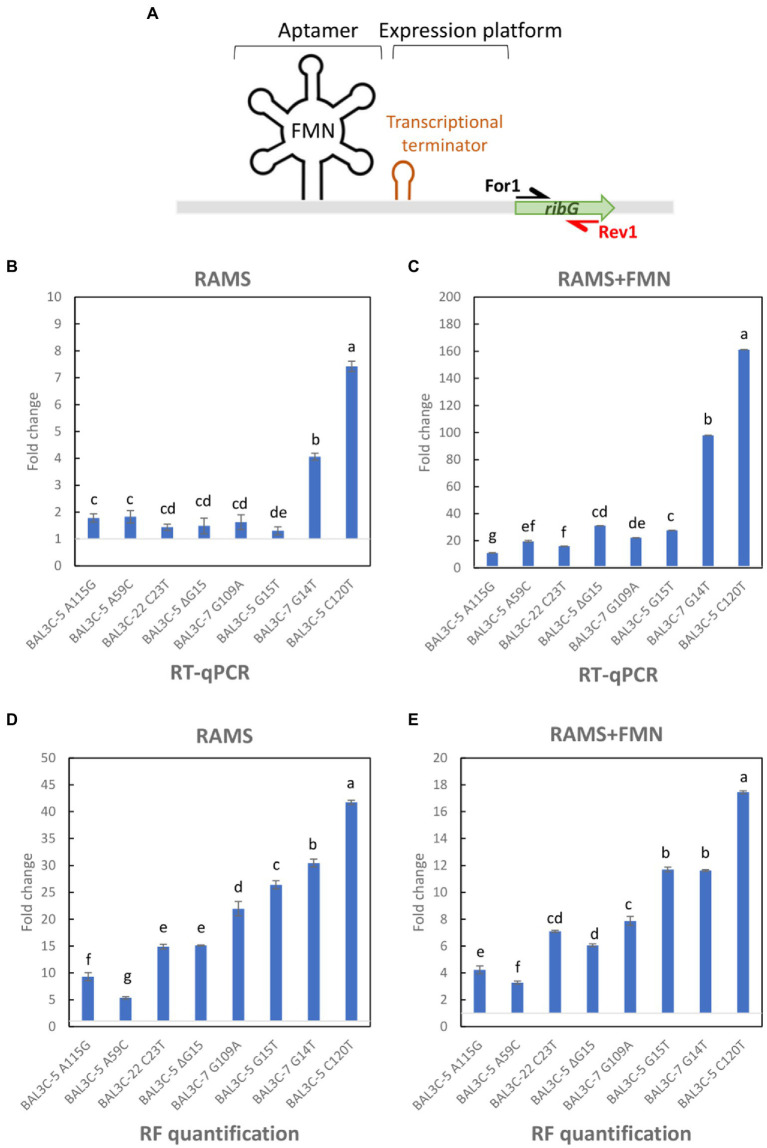
RT-qPCR analysis of *ribG* gene expression and evaluation of RF levels in *Weissella cibaria* cultures grown in RAMS or RAMS + FMN. **(A)** Schematic representation of the *rib* operon riboswitch including the FMN binding sensing aptamer and the expression platform in which a transcriptional terminator is formed in presence of FMN. The name and location of the primers used for the analysis are indicated. **(B,C)** Fold change of *ribG* gene expression in mutant strains compared with parental strains grown in RAMS **(B)** or RAMS + FMN **(C)**. **(D,E)** Fold change of RF production by mutant strains with regard to parental strains quantified from cultures submitted to RT-PCR in RAMS **(D)** or RAMS + FMN **(E)**. Different letters mean statistically significant difference (*p* ≤ 0.01).

The postulated mechanism of regulation of the *rib* operon expression made to predict that in the presence of FMN, this flavin will bind to the riboswitch aptamer and abortive transcription will take place, generating a transcript with its 3′-end at the ρ-independent terminator located upstream of *ribG* (Figures 1C, 7A).

To confirm that the different levels of transcription of the *rib* operon in the *W. cibaria* strains carrying mutant FMN riboswitches are related to the RF-overproducing phenotype, quantification of the *rib* mRNA levels was performed by RT-qPCR, and the changes in the expression of the first gene (*ribG*) of the *rib* operon in cultures grown in RAMS in the presence or absence of the effector FMN, were analyzed.

**Figure 8:**
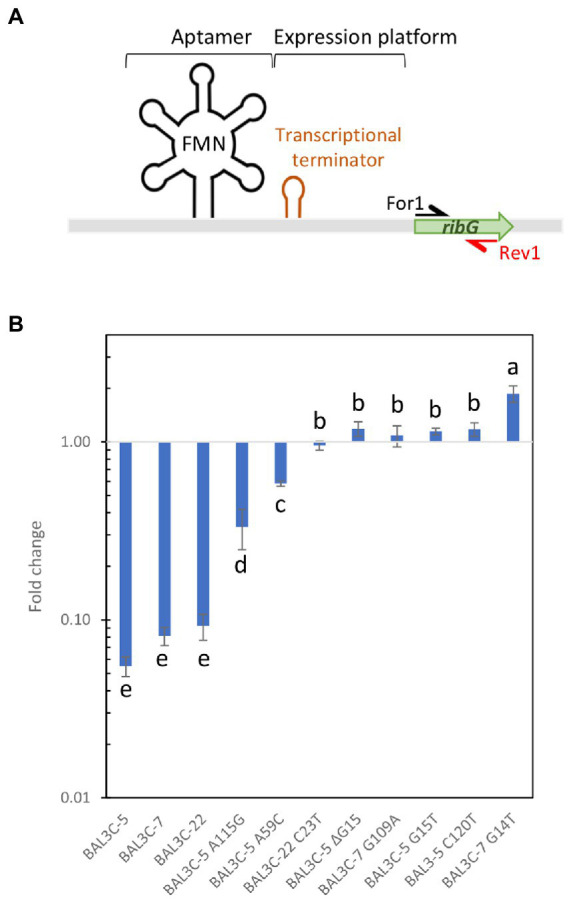
Analysis of the influence of FMN on transcription of the *ribG* gene in the *Weissella cibaria* parental and RF-overproducing strains. The bacteria were grown in RAMS and RAMS + 3 μM FMN media and, using total RNA preparations, cDNA was synthesized and employed as substrate to perform RT-qPCR analysis. **(A)** Schematic representation of the *rib* operon riboswitch. See details in legend of Figure 7. The name and location of the primers used for the analysis are indicated. **(B)** The fold change of mRNA levels in the presence vs. absence of FMN are represented for each strain. Different letters mean statistically significant difference (*p* ≤ 0.01).

To this end, total RNA preparations were used to generate *rib* cDNA, and a fragment of the *ribG* gene located downstream of the putative riboswitch transcriptional terminator was quantitatively amplified by qPCR using the For1 and For2 primers (Figure 7A). Mean Ct values were calculated and fold changes in expression between each mutant and its corresponding parental strain are depicted in Figures 7B,C. The results showed different levels of abundance depending on the strain analyzed and the growth medium used. All the mutants exhibited a statistically significant increase of *ribG* expression compared to their corresponding parental strain (*p* ≤ 0.05). The fold change values varied from 1.30 to 7.42 for mutant strains grown in RAMS (Figure 7B). In this case, BAL3C-5 C120T mutant strain showed the highest transcription level. A more pronounced increase of *ribG* expression was observed in cultures of the mutant strains compared to their parental strains when they were grown in RAMS + FMN. A 10.9- to 161.2-fold higher expression levels were observed for the mutant strains (Figure 7C). Under this condition, it was also found that the BAL3C-5 C120T strain had the highest expression level of *ribG*. When the ratio of *ribG* expression in the presence vs. absence of FMN was analyzed for each strain independently, it was observed that the wt strains presented a very low level of *ribG* expression (0.05–0.09-fold) in the presence of the FMN effector (Figure 8). Although not so pronounced, the BAL3C-5 A115G and the BAL3C-5 A59C mutant strains also showed a significant drop in transcript abundance when the RAMS + FMN growth medium was used (0.33- and 0.58-fold; Figure 8), indicating that expression of the *rib* operon was still partially repressed by FMN-riboswitch aptamer interactions. This was not the case for the rest of the mutant strains, which presented a similar expression in the presence or absence of FMN in the growth medium (from 0.95- to 1.18-fold), with no significant statistical differences, beside BAL3C-7 G14T, which showed a slight but significant 1.86-fold higher level in RAMS + FMN medium than in RAMS medium (Figure 8), results that supported absence of post-transcriptional regulation mediated by the FMN effector.

In parallel, total RF concentration from cultures submitted to RT-qPCR was also evaluated. The RF production was expressed, as in the case of the RT-qPCR data depicted in Figures 7B,C, as fold change detected in the mutants with regard to their parental strains in RAMS (Figure 7D) and RAMS + FMN (Figure 7E). The results revealed, as shown in Table 3, that all the mutants produced statistically significant higher levels of RF in the two media tested (*p* ≤ 0.05). Furthermore, the enhancement of production ranged from 5.3- to 41.7-fold for cultures grown in RAMS and from 3.2- to 17.4-fold for cultures grown in RAMS + FMN. In addition, in both media tested, BAL3C-5 A59C exhibited the lowest fold change value in comparison with the rest of the mutants and BAL3C-5 C120T showed the highest fold change in RF levels.

### 3.5. Expression profiling of the riboswitch region in presence and absence of FMN

Expression of the FMN riboswitch aptamer was quantified at the level of mRNA abundance. The aim of this analysis was to determine potential changes in the transcription of the untranslated lider region of the *rib* operon, upstream of the putative transcriptional terminator. Transcriptional analysis was carried out as above from cultures grown in both RAMS and RAMS + FMN, but using primers located upstream (For2) and within the aptamer (Rev2) for amplification during qPCR analysis (Supplementary Figure S7A). No statistically significant differences in mRNA levels between parental and mutant strains were observed (Supplementary Figure 7B), when cultures were grown in RAMS were analyzed. These results were expected, since nucleotide changes present in the mutant strains should not affect the transcriptional initiation signals of the *rib* operon. However, when cultures were grown in RAMS + FMN, the detected levels of the transcripts were significantly lower for the mutants compared with their corresponding parental strains (Supplementary Figure 7B). In addition, transcript abundance in the mutants showed a range of variation from 0.27- to 0.83-fold lower Ct values than that of the parental strains. Consequently, the overall RT-qPCR analysis indicated that, as expected, the untranslated leader region of the *rib* mRNA has a different fate to that of the coding one in both the parental and the mutant strains.

### 3.6. Determination of the complete DNA sequence of the chromosome of BAL3C-5 and BAL3C-5 C120T strains

**Figure 9:**
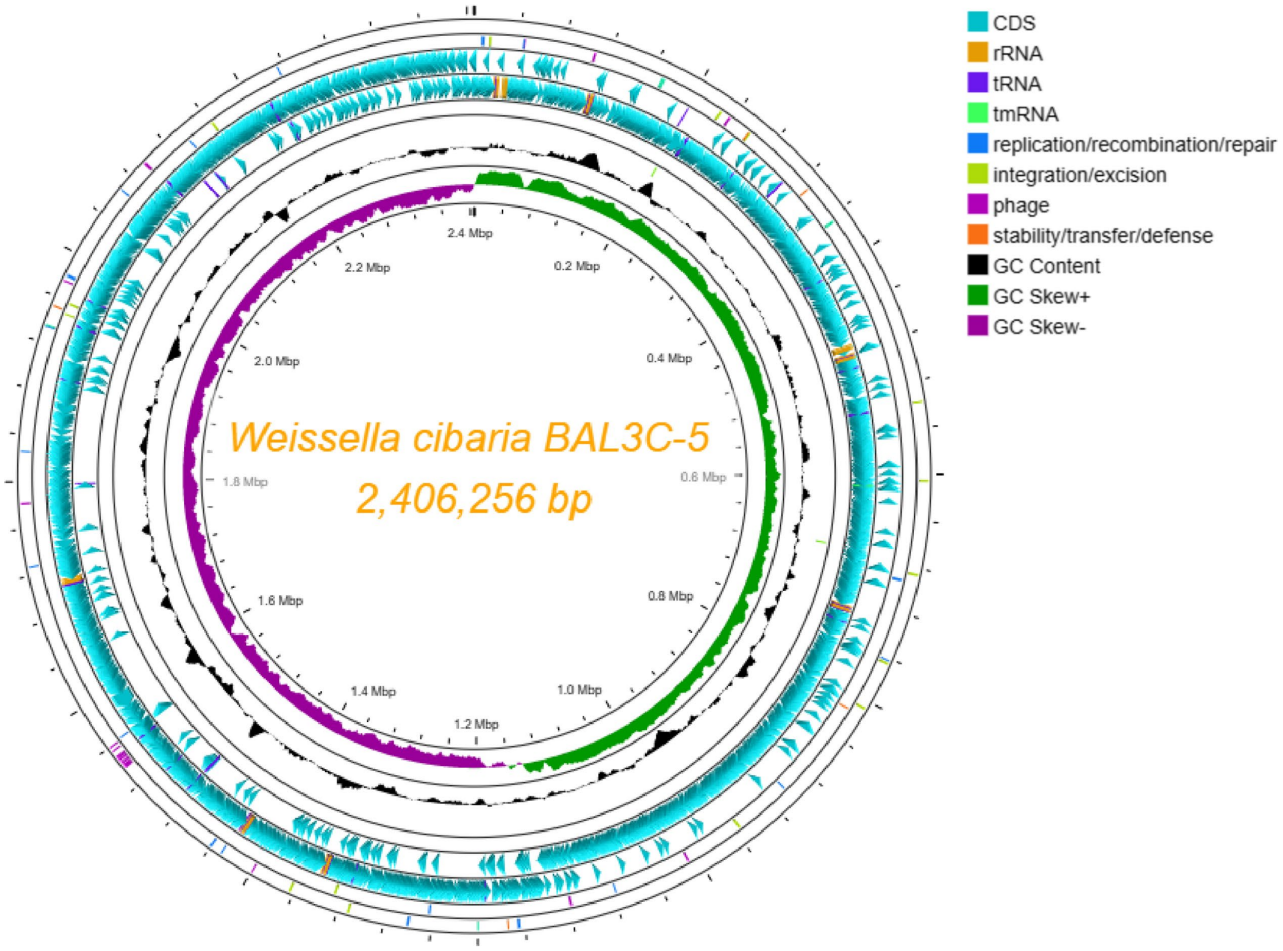
Circular genome representation of *Weissella cibaria* BAL3C-5. Each element color of each circle is shown in the legend. The different rings provide information about: forward CDS, reverse CDS, GC Skew, etc. rRNA, tRNA, and tmRNA are located in the same ring of CDS.

Since BAL3C-5 C120T possesses the highest RF-overproducing phenotype among the studied strains, it was chosen, together with the parental BAL3C-5 strain, to carry out the sequencing of their genomes. A total of 83,974 (BAL3C-5) and 115,954 (BAL3C-5 C120T) mean raw reads comprising 397.4 and 473.2 Mb were obtained, indicating mean assembly coverage of 160X and 200X, respectively. Assembly resulted in 1 contig, with 2,406,256 bp of genome size and a GC% content of 45.15% for both strains. Annotation using prokka 1.14.6 revealed a total of 2,350 genes, distributed in 2,233 CDS, 88 tRNA, 28 rRNA and 1 tmRNA. Genome visualization is shown in Figure 9. The size of the circular chromosome of both stains was in accordance with the 13 complete genomes of *W. cibaria* available in the NCBI database which range between 2,3 and 2,6 Mbp. Moreover, after comparing the genomes of the wt and the mutant strains analyzed only a single mutation was detected at position 446,494, which corresponds to the C120T alteration in the riboswitch of the mutant strain. These sequences were deposited in GenBank under the accession numbers CP116386 (BAL3C-5) and CP116385 (BAL3C-5 C120T).

The genomes were also screened against coding genes of antibiotic resistance and virulence factors. Antibiotic resistance evaluation using the CARD database confirmed that *W. cibaria* BAL3C5 and BAL3C5 C120T genomes did not harbor any specific resistance genes. In the same way, when virulence factors determination was carried out, Island4viewer software showed the absence of pathogen-associated genes, homologs of resistance genes, curated resistance genes, homologs of virulence factors and curated virulence factors (data not shown). Thus, safety parameters evaluated *in silico* support the potential use of the strain C120T for the development of functional foods.

## 4. Discussion

### 4.1. A strategy for identification of RF-overproducing strains

The FMN riboswitch regulatory element of the *rib* operon is composed of a FMN sensing domain and of the expression platform. It is thought that this RNA riboswitch presents two different conformations corresponding to the “OFF state” or FMN-bound state which facilitates the formation of the riboswitch aptamer and of a ρ-independent transcriptional terminator, and the “ON state” in which an anti-terminator structure is formed in the absence of FMN enabling the transcription of the *rib* operon (Vitreschak et al., 2002; Winkler et al., 2002 and Figure 1C). Moreover, roseoflavin-resistant strains usually harbor mutations in the riboswitch, which may lead to a reduction of production of RF in the presence of FMN.

Thus, in the present study a strategy for the *in vitro* identification and selection of mutant strains from *W. cibaria* species has been examined. Exposure to the selective pressure of high roseoflavin concentrations followed by the sequencing of the corresponding FMN riboswitch coding sequences of the resulting treated cultures was explored as an approach for the detection and selection of high RF-overproducing strains. The DNA sequencing of the *rib* operon leader region of the roseoflavin-treated cultures revealed a significant number of mutations (G14T, G15T, T16G, C23T, A59C, G87A, G109A, A115G, and C120T) and one deletion (ΔG15) located at the FMN riboswitch. The sensor domain of the FMN riboswitch is an aptamer, which contains five hairpins (from P2/L2 to P6/L6; P known as helix and L known as loop) and a P1 helix as the base of this element, which is predicted to be formed in the *rib* mRNA and interact with FMN (Figure 5). All the detected mutations were positioned in the aptamer and most of them were in peripheral locations, with P2 (containing positions 14, 15, 16, and 23) and P6 helices (containing positions 109, 115 and 120) being special hot-spots harboring most of the mutations. Only mutations A59C and G87A were located outside of the stem-loop structures. When comparing the position of these mutations with those detected in previous studies (Burgess et al., 2004, 2006; Serganov et al., 2009; Ripa et al., 2022), it was observed that most of them belong to conserved nucleotides among distant species (Figure 5), such as *B. subtilis, B. amyloliquefaciens, Fusobacterium nucleatum, L. mesenteroides, L. lactis*, *Propionibacterium freudenreichii.* or *L. plantarum.* Therefore, the location of these mutations may indicate that they are responsible for the RF-overproducing phenotype. According to the crystallographic study performed by Serganov et al. (2009) on the FMN riboswitch of *F. nucleatum* in the presence of FMN, the P2/L2 and P6/L6 structures as well as P3/L3 and P5/L5 interact with each other forming a tertiary structure. Consequently, mutations in these regions may lead to deregulation and overproduction of RF. Taking the model of the FMN riboswitch of Serganov et al. (2009), the proposed interactions between the nucleotides in the *W. cibaria* riboswitch are shown in Figure 5. In this regard, it is expected that the G115 ribonucleotide would interact with the ribonucleotide G17. The ribonucleotide C109 (together with C108) could interact forming a triplet with C29/G30 and G87, which are thought to interact with the phosphate group of the FMN. Also, ribonucleotides adjacent to C120 (whose mutation leads to the highest RF production) such as G118/T119 should interact with A26/C27 in the presence of FMN forming a tertiary structure. Consequently, mutations in these key positions may also be responsible for the overproducing phenotypes observed.

When the folding of the aptamer of each mutation was predicted with the RNA fold program, it was observed that some mutations resulted in conformational changes of the complex secondary structure (Figure 4). This was the case of ΔG15, G14U and G15U changes at the mRNA level, which may have an impact on the stability of the riboswitch aptamer, and thus, in the overproduction of RF. Moreover, the ΔG of each resulting secondary structure was also analyzed. The lower the thermodynamic energy of the structure, the more structured and stable it should be. Thus, the mutants ΔG15, G14T, G15T, C23T, C120T showed a higher value, accordingly a lower stable structure was expected. Indeed, these mutants, together with G109A (which takes part in key interactions in the riboswitch) were the higher RF producers. By contrast, the structures derived from A59C and A115G mutations, showed the same ΔG energy as the parental strains and they produced the lowest concentrations of RF, compared with the other mutants. These results show, as expected, that the mechanistic reason for an RF-overproducing phenotype is complex and diverse, since nucleotide mutations located at the riboswitch aptamer could affect interaction with other nucleotides/helices, and they could also lead to different secondary structures with different thermodynamic energy. Taking in account these features, sequence and folding structure analysis may be considered as tools for tentative prediction of overproducing phenotypes prior the *in situ* quantification of RF producing abilities and even isolation of mutant strains as shown in this work.

### 4.2. Selection and evaluation of RF-overproducing spontaneous mutants from RF-and dextran-producing *Weissella cibaria* populations

Once the mutations corresponding to the roseoflavin-treated cultures were detected *in vitro,* the next step was the selection of the mutants of interest. This approach was carried out by culture plating taking advantage of the EPS-producing capacity of the treated strains, and assuming that the overproducing phenotype would give the colonies a yellow color. This was evident when the LAB were plated in the presence of sucrose due to the large mucous colonies generated in which the yellow color was more amplified compared with the small colonies devised in the presence of glucose. With this strategy, we were able to recover and select the cited mutants. When the growth and RF-overproducing capabilities were analyzed in real time, no differences in growth performances were observed between the parental and their mutant derivatives. Hence, growth was not affected by the mutations detected neither the overproduction of RF. The mutant BAL3C-5 C120T showed the greatest overproduction phenotype while mutant BAL3C-5 A59C the least. Regarding the regulatory capacity of FMN or RF, it was observed that the mutant cultures presented an apparent production of RF independent of the presence of the flavins in the growth medium (Figure 6A), but not the parental strains in which the production of vitamin B_2_ was delayed upon growth in RAMS + RF and RAMS + FMN, and reduction of the flavin levels was observed at the beginning of the bacterial growth (Figure 6B). Thus, it was confirmed that mutant strains could produce RF without consuming flavins present in the medium, and RF production in mutant strains seemed to be deregulated. In addition, it has been reconfirmed that the fluorescent detection of RF in real time described by us (Mohedano et al., 2019) is suitable for real-time quantification of the vitamin production.

Evaluation of the RF production in RAM and RAMS after 16 h of growth gave again the same results, and the same pattern of vitamin production by the mutants, with strain BAL3C-5 C120T being the highest RF overproducer and with no significant differences in viable cells (Table 3). The bacterial cultures showed further growth in RAMS, due to the fact that the RAM medium contains 2% glucose, whereas in RAMS, an additional 2% sucrose was also present. This fact would also support the higher RF production in RAMS compared with RAM. Another feature that should be highlighted is that the mutant strains externalize most of the RF produced. Given that overproduction of RF has no beneficial effects on mutant growth (Figure 4A), a possible explanation for the observed behavior is that high excess of unneeded RF in the cytosol of the mutants is released to the environment by active transport and/or diffusion to avoid toxic effects. In addition, independently of the mechanism, this release is a very desirable characteristic considering a possible application in the *in situ* biofortification of different fermented foods. Thus, in the present study the most promising *W. cibaria* BAL3C-5 C120T mutant strain was able to generate 6.70 mg/l extracellular RF.

Recently, we have described the selection, from BAL3C-5, BAL3C-7, and BAL3C-22, of three mutant strains named as BAL3C-5 B2, BAL3C-7 B2, and BAL3C-22 B2 (Hernández-Alcántara et al., 2022), and renamed in the present study as BAL3C-5 G15T, BAL3C-7 G109A, and BAL3C-22 C23T strains, respectively. Among them, the highest producer, BAL3C-5 B2, showed synthesis of RF in concentrations up to 3.40 mg/L. Similar RF production has been obtained in the current work with this mutant (4.16 mg/L), which is 1.6-fold lower concentration than that observed with BAL3C-5 C120T (6.67 mg/L). Furthermore, to assess the RF-overproducing phenotype of BAL3C-5 C120T in a wider context, it was also compared with others RF-overproducing LAB obtained after roseoflavin treatment. This RF production of the mutant was higher than the maximum amount produced by previously obtained LAB mutant derivatives. Accordingly, it was found that *Lactobacillus fermentum* PBCC11 was able to produce approximately 1.20 mg/l (Russo et al., 2014), while this concentration dropped drastically to 0.90 mg/L for *L. lactis* (Burgess et al., 2004) and just about 0.60 mg/L in the case of *L. mesenteroides* and *L. plantarum* (Burgess et al., 2004; Capozzi et al., 2011). In addition, the ability of BAL3C-5 C120T strain was even higher than that of the recently identified high RF-overproducing *L. plantarum* strains showing 1.30–3.7 mg/L (Juarez del Valle et al., 2014; Mohedano et al., 2019; Yépez et al., 2019; Ripa et al., 2022). Recently, the characterization of two LAB species with high RF production capability has been carried out. Kim et al. (2021) highlighted the RF-overproducing phenotype of the *L. plantarum* HY7715 isolated from kimchi, selected under roseoflavin pressure, which was able to produce up to 14.50 mg/L. In the same way, Spacova et al. (2022) described a novel human isolate *L. reuteri* AMBV339, which showed a high natural RF overproduction of 18.16 mg/l. In both studies, it was also stated the resulting biomass of each strain after the RF production. Therefore, when we analyzed the RF concentration in reference to the biomass, considering the detected viable cells (CFU/L), the strain *W. cibaria* BAL3C-5 C120T showed the highest total RF production related to viable cells, since 1.41 × 10^11^ CFU/ml produced ≈ 6.78 mg/L, whereas approximately 10- or 60-fold higher biomass (1.55×10^12^ or 6 × 10^12^ CFU/L) of *L. plantarum* HY7715 or *L. reuteri* AMBV339 were required to produce ≈ 14.5 or 18.16 mg/L. Thus, it is expected that if we produce 10-fold higher biomass, we can reach levels of RF production of ≈ 67.8 mg/L. In addition, to our knowledge, and among the *W. cibaria* strains identified so far, *W. cibaria* BAL3C-5 C120T is the highest RF-overproducing strain currently available.

In addition, the determination of the DNA sequence of the BAL3C-5 C120T has revealed that a single change in the genome is solely responsible for an increase in RF production, which rose from 0.1 mg/L in the parental strain to almost 7 mg/L in the mutant strain. As far as we know this is the first time, that it has been certified that a single alteration in the genome is responsible for such a phenotypic change in RF production. Taking into account both its RF-overproducing and its dextran producing phenotypes, strain BAL3C-5 C120T has great potential in the production of *in situ* biofortified foods. Through this strategy, fermented foods with improved nutritional and functional properties, as well as suitable rheological and structural characteristics, could be developed. This supposes a great potential and interest of what this species can offer. In this regard, a first approach was performed by us (Hernández-Alcántara et al., 2022) in the development of experimental biofortified breads which may result of great interest for the manufacturing of functional breads. Indeed, *Weissella* genus include strains that are frequently present in spontaneously fermented food, among them *W. cibaria* strains are frequently isolated from sorghum. Furthermore, many *Weissella* strains have shown probiotic and biotechnological properties of interest for the food industry, but some clinical isolates belonging to this genus have been also isolated (Fusco et al., 2015). For this reason, currently none of the *Weissella* species has the *Qualified Presumption* of Safety (QPS) or the Generally Recognized as Safe (GRAS) status. Consequently, they have not been yet used as a starter or co-culture by the food industry. Nevertheless, due to the interest of these LAB, currently evaluation of the probiotic and safety properties of *Weissella* strains are investigated at the phenotypical and comparative genomic levels with the aim to identify potential starter or co-adjuvant strains for food fermentations (Falasconi et al., 2020; Apostolakos et al., 2022).

In this context, it has to be stated that although analysis of the genome of BAL3C-5 C120T did not reveal the existence of genes encoding virulent factors or resistance to antibiotics, before utilization of this *W. cibaria* strain for production of biofortified bread, it will be necessary to asses experimentally its safety status. Moreover, for the potential use of this strain to develop other types of fermented food, in which the bacteria will be alive, it will be necessary to assess his probiotic potential.

### 4.3. Transcriptional insights on RF production and expression profile comparison between mutants and their parental strains

After evaluating the different RF production of the selected mutants, we attempted to analyze its production at the transcriptional level and elucidate potential changes in the expression of *ribG*, the first gene in the *rib* operon. In the absence of FMN addition in the growth medium, most of the mutants presented only a slight increase of expression (1.3–1.8-fold) with respect to the corresponding parental strain, except the BAL3C-7 G14T and BAL3C-5 C120T strains, which presented a clearly higher expression (4.0- and 7.4-fold) than the rest of the mutant and wt strains (Figure 7B). The same type of results was observed in presence of FMN, although *ribG* gene expression levels in mutants compared with their respective parental strains were much higher (Figure 7C), BAL3C-7 G14T and BAL3C-5 C120T reaching values of 98 and 161-fold increase, respectively. This enhanced difference seems to be due to the low expression levels of *ribG* in the wt strains in presence of FMN, a conclusion inferred from the fact that the levels of transcription in the presence vs. in absence of FMN ranged from 0.05- to 0.09-fold for the 3 wt strains (Figure 8). When correlating the *ribG* expression and the RF production data, except for the two main producers (BAL3C-7 G14T and BAL3C-5 C120T strains), the order of transcript abundance did not match perfectly with RF synthesis measured in culture medium, since the production levels were much higher than those observed at the transcriptional level. Thus, other factors in the synthesis of RF should be considered. First, only the expression of the *ribG* has been evaluated and not of the downstream genes (*ribB*, *ribA*, and *ribH*) of the *rib* operon, whose products are also involved in the RF synthesis. For example, changes in the untranslated region of the *rib* operon might influence the folding of the total transcript, affecting its half-life and internal processing due to endoribonucleases. In addition, mRNA turnover rate for each strain may have also changed. Indeed, mRNA stability and the rate at which each mRNA is degraded and/or translated are important factors for gene expression control (Cooper, 2000). These features may have also varied in the mutant strains and although transcription remains as the main level where gene expression is regulated, changes in mRNA degradation rate also have great influence in controlling the transcript levels, and subsequently protein levels, and finally, in this case, the RF production levels.

When comparing the behavior of the BAL3C-5, BAL3C-7, and BAL3C-22strains in the RAMS and RAMS + FMN environments (Figure 8), it was clear that the expression of *ribG* gene was almost insignificant in the presence of FMN. Thus, production of RF in the three wt strains seems to be properly regulated. However, in the case of BAL3C-5 A59C and BAL3C-5 A115G strains, which also showed lower transcript abundance in RAM + FMN grown cultures, *ribG* expression was partially regulated as they did not reach such an expression decay as that in the wt strains. It should be emphasized that both BAL3C-5 A59C and BAL3C-5 A115G showed the lowest RF production levels among all mutant strains. Furthermore, the Gibbs free energy of their FMN riboswitch aptamer was identical to that of the parental strains. Thus, the stability of their structures probably had not been compromised and therefore, the regulation in RF production would take place, at least partially due to the corresponding mutations, which could be the cause of the low levels of RF production compared to the rest of the mutants. Regarding to the remaining mutants, a similar expression was observed independent of the presence of FMN. These strains seem to be no longer subjected to regulatory response, which should lead to a RF production independent of FMN concentration. If attention is paid to the stability of the aptamer structures of the isolated strains (Figure 4), it can be seen that all of them (except the BAL3C-7 G109A strain) present less stability than their parental strains. This may be a possible explanation of the total deregulation in the production of RF, as the position of the mutation may be the main cause for the different levels of production observed. The BAL3C-7 G109A strain appears to have a more stable regulatory structure (according to its ΔG) than that of BAL3C-7, with the location of the mutation in a predicted key position for interaction of the phosphate group of the FMN with the riboswitch, it nevertheless results in a constitutive production of the vitamin that does not respond to transcriptional regulation by FMN.

Once the results corresponding to the expression of *ribG* gene were analyzed, it was decided to investigate the situation of expression of the FMN riboswitch region. Transcriptional analysis showed no significant differences between mutant strains in the absence of FMN (Supplementary Figure S7). However, when the bacterial cultures were grown in RAMS + FMN, differences were detected (Supplementary Figure S7). Under this condition, mutant strains presented lower abundance of transcript compared to their parental strains. A feasible hypothesis is that in the presence of FMN, in the case of the wt strains, the aptamer would be formed and stabilized by the binding of the flavin, a situation that could give greater stability to the leader region of the *rib* mRNA, since, as it has been observed in other studies (Richards and Belasco, 2021). The binding of the ligand to the riboswitch aptamer would be protecting the RNA from degradation by blocking the access of endo- and exo-nucleases to regions of the riboswitch susceptible to being attacked. On the contrary, in the case of the mutants, where it is predicted that the binding of FMN will be negligible or decreased, although the stability of the aptamer structure will be lower, it could still be formed and processing of the riboswitch aptamer and adjacent regions by nucleases could take place. Therefore, this could be the reason why a lower transcript signal is detected in mutants.

In this regard, those with the highest expression in the presence of FMN (apart from the wt strains), were BAL3C-5 A59C and BAL3C-5 A115G. These results correlated with the data previously observed. These are the mutant strains that showed lower *ribG* expression in the presence of FMN, the consequence of a partially regulated expression (Figure 8). These two bacteria are, among mutants, the strains with the highest expression of the regulatory region and could indicate stabilization of the riboswitch structure. Therefore, the formation of the A59C and the A115G aptamers would indicate a greater stability and abundance of transcript as well as more regulation compared with the rest of the mutants and thus, less RF production. On the contrary, the strain with the lowest expression carried the change G109A, mutation at a position that it has been previously observed to be key for the interaction with the FMN.

## 5. Conclusion

The method described here was found to be a suitable strategy for selecting spontaneous riboflavin-overproducing and dextran-producing mutants of *W. cibaria*. It has been possible to observe significant differences at the transcriptional level between the different strains, confirming the increase in *ribG* transcription in the highest overproducing strains compared to the rest of the strains. In this regard, it must be highlighted the selection of the BAL3C-5 C120T strain, as the highest RF overproducer. Above all, it has been ascertained that a single alteration in the genome is responsible for such a phenotypic change. Moreover, in the future, after evaluation of its probiotic potential and confirmation of its safety status of the BAL3C-5 C120T strain, new perspectives will be opened regarding the characterization of its potential use in the food and health industries, as an interesting strategy for the biofortification of potentially functional foods.

## Data availability statement

The datasets presented in this study can be found in online repositories. The names of the repository/repositories and accession number(s) can be found at: https://www.ncbi.nlm.nih.gov/genbank/, CP116386 and https://www.ncbi.nlm.nih.gov/genbank/, CP116385.

## Author contributions

MD and PL: conceptualization. ID-O, MM, and PL: methodology. ID-O, JR-M, and GdS: software. ID-O, LM-L, and MM: investigation. ID-O and PL: data curation. ID-O, MM, and PL: writing—original draft preparation. GdS, MD, MT, and PL: writing—review and editing. MD, MM, and PL: supervision. GdS, PL, and MD: funding acquisition. All authors contributed to the article and approved the submitted version.

## Funding

This research was funded by the Spanish Ministry of Science, Innovation and Universities (grant RTI2018-097114-B-I00), CSIC (grant COOPA20488), the University of Basque Government (grants IT1662-22 and PIBA_2020_1_0032), and the University of Basque Country (UPV-EHU; GIU19/014). ID-O is the beneficiary of a postdoctoral grant Margarita Salas by UPV-EHU (MARSA21/25) in the framework of “the requalification of the Spanish university system” funded by the European Union-Next Generation EU.

## Conflict of interest

The authors declare that the research was conducted in the absence of any commercial or financial relationships that could be construed as a potential conflict of interest.

## Publisher’s note

All claims expressed in this article are solely those of the authors and do not necessarily represent those of their affiliated organizations, or those of the publisher, the editors and the reviewers. Any product that may be evaluated in this article, or claim that may be made by its manufacturer, is not guaranteed or endorsed by the publisher.

## Footnotes

^1^https://usegalaxy.org

^2^https://proksee.ca

^3^https://www.pathogenomics.sfu.ca/islandviewer/
