# Supplementary material for: A methodology for the selection and characterization of riboflavin-overproducing Weissella cibaria strains after treatment with roseoflavin
Source: Front Microbiol. 2023 Apr 6;14:1154130. doi: 10.3389/fmicb.2023.1154130 (PMC10116070; doi:10.3389/fmicb.2023.1154130)
Supplement: Supplementary file 1 [file Data_Sheet_1.docx]

Supplementary Material

**A methodology for the selection and characterization of riboflavin-overproducing *Weissella cibaria* strains after treatment with roseoflavin**

**Iñaki Diez-Ozaeta^1,2^, Lucía Martín-Loarte^1^, Mari Luz Mohedano^1^, Mercedes Tamame^3^, José Ángel Ruiz-Masó^1^, Gloria del Solar^1^, Paloma López^1*^ and María Teresa Dueñas^2*^**

^1^Departamento de Biotecnología Microbiana y de Plantas. Centro de Investigaciones Biológicas Margarita Salas (CSIC), Madrid, Spain.

^2^Departamento de Química Aplicada, Facultad de Química, Universidad del País Vasco (UPV/EHU), San Sebastián, Spain.

^3^Instituto de Biología Funcional y Genómica, (IBFG) CSIC-Universidad de Salamanca, Salamanca, Spain.

***Correspondence:** plg@cib.csic.es (Paloma López), mariateresa.duenas@ehu.eus (Mª Teresa Dueñas)

**Keywords:** *Weissella cibaria*, lactic acid bacteria, vitamin B2 (riboflavin), FMN riboswitch, roseoflavin, riboflavin overproducing bacteria, regulation of rib operon

**A**

BAL3C-5

BAL3C-7

BAL3C-22

BAL3C-5 A59C

BAL3C-5 A115G

BAL3C-5 ΔG15

BAL3C-22 C23T

BAL3C-7 G109A

BAL3C-5 G15T

BAL3C-7 G14T

BAL3C-5 C120T

RAM medium

**B**

**C**

**D**

BAL3C-5

BAL3C-5 C120T

BAL3C-5 and BAL3C-5 C120T

**Supplementary Figure S1. Photographs of *W. cibaria* strains grown in liquid (A) and solid (B, C, D) media. A**. The *Weissella cibaria* BAL3C-5, BAL3C-5 and BAL3C-22 parental wild-type strains as well as the indicated riboflavin-overproducing mutant strains were grown in RAMS (riboflavin assay medium, Difco supplemented with 2% sucrose) medium. **B**. Mucous white colonies of the BAL3C-5 strain grown on petri dish containing MRSS (MRS supplemented with 2% sucrose) medium. **C**) Mucous yellow colonies of the BAL3C-5 C120T riboflavin-overproducing strain grown on a petri dish containing MRSS. **D.** Mixed culture of BAL3C-5 (with colonies) and BAL3C-5 C120T mutant (yellow colonies) strains grown on a petri dish containing MRSS.

BAL3C-5

BAL3C-5 A59C

BAL3C-5 A115G

BAL3C-5 ΔG15

BAL3C-5 G15T

BAL3C-5 C120T

BAL3C-7

BAL3C-7 G109A

BAL3C-7 G34T

BAL3C-22

BAL3C-22 C23T

BAL3C-5

BAL3C-5 A59C

BAL3C-5 A115G

BAL3C-5 ΔG15

BAL3C-5 G15T

BAL3C-5 C120T

BAL3C-7

BAL3C-7 G109A

BAL3C-7 G34T

BAL3C-22

BAL3C-22 C23T

**Supplementary Figure S2. Statistical analysis of total RF production by the *W. cibaria* parental and RF-overproducing strains grown in RAMS medium.** Upper panel comparison of mutant strains, with the corresponding parental strain. Lower panel, comparison of all the strains analysed. Values with different letters or strains grouped within a bracket (**) indicate significant differences (*p*≤0.05).

BAL3C-5

BAL3C-5 A59C

BAL3C-5 A115G

BAL3C-5 ΔG15

BAL3C-5 G15T

BAL3C-5 C120T

BAL3C-7

BAL3C-7 G109A

BAL3C-7 G34T

BAL3C-22

BAL3C-22 C23T

BAL3C-5

BAL3C-5 A59C

BAL3C-5 A115G

BAL3C-5 ΔG15

BAL3C-5 G15T

BAL3C-5 C120T

BAL3C-7

BAL3C-7 G109A

BAL3C-7 G34T

BAL3C-22

BAL3C-22 C23T

**Supplementary Figure S3. Statistical analysis of total RF production by the *W. cibaria* parental and RF-overproducing strains grown in RAM medium.** Upper panel comparison of mutant strains, with the corresponding parental strain. Lower panel, comparison of all the strain analysed. Values with different letters or strains grouped within a bracket (**) indicate significant differences (*p*≤0.05).

BAL3C-5

BAL3C-5 A59C

BAL3C-5 A115G

BAL3C-5 ΔG15

BAL3C-5 G15T

BAL3C-5 C120T

BAL3C-7

BAL3C-7 G109A

BAL3C-7 G34T

BAL3C-22

BAL3C-22 C23T

BAL3C-5

BAL3C-5 A59C

BAL3C-5 A115G

BAL3C-5 ΔG15

BAL3C-5 G15T

BAL3C-5 C120T

BAL3C-7

BAL3C-7 G109A

BAL3C-7 G34T

BAL3C-22

BAL3C-22 C23T

**Supplementary Figure S4. Statistical analysis of free RF production by the *W. cibaria* parental and RF-overproducing strains grown in RAMS medium.** Upper panel comparison of mutant strains, with the corresponding parental strain. Lower panel, comparison of all the strains analysed. Values with different letters or strains grouped within a bracket (**) indicate significant differences (*p*≤0.05). fferences (p≤0.05).

BAL3C-5

BAL3C-5 A59C

BAL3C-5 A115G

BAL3C-5 ΔG15

BAL3C-5 G15T

BAL3C-5 C120T

BAL3C-7

BAL3C-7 G109A

BAL3C-7 G34T

BAL3C-22

BAL3C-22 C23T

BAL3C-5

BAL3C-5 A59C

BAL3C-5 A115G

BAL3C-5 ΔG15

BAL3C-5 G15T

BAL3C-5 C120T

BAL3C-7

BAL3C-7 G109A

BAL3C-7 G34T

BAL3C-22

BAL3C-22 C23T

**Supplementary Figure S5. Statistical analysis of free RF production by the *W. cibaria* parental and RF-overproducing strains grown in RAM medium.** Upper panel comparison of mutant strains, with the corresponding parental strain. Lower panel, comparison of all the strains analysed. Values with different letters or strains grouped within a bracket (**) indicate significant differences (*p*≤0.05).

BAL3C-5

BAL3C-5 A59C

BAL3C-5 A115G

BAL3C-5 ΔG15

BAL3C-5 G15T

BAL3C-5 C120T

BAL3C-7

BAL3C-7 G109A

BAL3C-7 G34T

BAL3C-22

BAL3C-22 C23T

BAL3C-5

BAL3C-5 A59C

BAL3C-5 A115G

BAL3C-5 ΔG15

BAL3C-5 G15T

BAL3C-5 C120T

BAL3C-7

BAL3C-7 G109A

BAL3C-7 G34T

BAL3C-22

BAL3C-22 C23T

**Supplementary Figure S6. Statistical analysis of dextran produced by the *W. cibaria* parental and RF-overproducing strains grown in RAMS medium.** Upper panel comparison of mutant strains, with the corresponding parental strain. Lower panel, comparison of all the strains analysed. Values with different letters or strains grouped with a bracket (** or *) indicate significant differences (*p*≤0.05) among them. n.s (not significant).

**Supplementary Figure S7. RT-qPCR analysis of FMN riboswitch region. A**. Schematic representation of the *rib* operon riboswitch including the FMN binding sensing aptamer and the expression platform in which a transcriptional terminator is formed in presence of FMN. Ratio of expression of the FMN riboswitch of mutant strains compared with that of parental strains in cultures grown in RAMS (**B**) or RAMS+FMN (**C**).
